# Ambivalent attitudes promote support for extreme political actions

**DOI:** 10.1126/sciadv.adn2965

**Published:** 2024-06-12

**Authors:** Joseph J. Siev, Richard E. Petty

**Affiliations:** ^1^ Department of Psychology, The Ohio State University, Columbus, OH 43210, USA.; ^2^ Darden School of Business, University of Virginia, Charlottesville, VA 22901, USA.

## Abstract

Political extremism varies across people and contexts, but which beliefs will a person support through extreme actions? We propose that ambivalent attitudes, despite reducing normative political actions like voting, increase support for extreme political actions. We demonstrate this hypothesized reversal using dozens of measures across six studies (*N* = 13,055). The effect was robust to relevant covariates and numerous methodological variations and was magnified when people’s attitudinal or ideological positions were more polarized. It appears to occur because being conflicted about political issues can feel psychologically uncomfortable, making extreme actions more appealing. Notably, this emerged when people thought ambivalence was justified, whereas leading them to consider ambivalence unjustified suppressed the effect, suggesting that ambivalent people are coping with but not necessarily trying to reduce their ambivalence. These results highlight the interplay of affective and cognitive influences in extreme behavior, showing that beliefs people feel justifiably conflicted about can promote extremism.

## Teaser

Despite reducing normative forms of political action, greater ambivalence predicts more support for extreme political actions.

## INTRODUCTION

Despite heightened political polarization, people still commonly feel conflicted about their political beliefs and attitudes (*1*–*2*). Fittingly, previous research identifies mixed implications of attitudinal ambivalence (*3*–*5*) for political behavior. On the one hand, ambivalence can reduce partisan biases and promote more thoughtful, deliberative political decision-making (*1*, *6*). On the other hand, ambivalence tends to reduce voting and political participation more broadly (*7*–*9*). Beyond these established findings, in the current research, we present evidence for a previously unrecognized but potentially important consequence of experiencing attitudinal conflict about political issues: greater support for extreme forms of political action.

Following previous work [e.g., (*10*–*11*)], we define behaviors as more extreme the more they deviate from normative action in a particular domain or context. Supporting such actions can be an effective way of demonstrating strong attitudinal commitment. For example, when the domain is “political advocacy” (i.e., pro-attitudinal political behavior), common forms of political advocacy like voting are not extreme, but unusually strong forms of activism like violence against partisan opponents would be extreme. Or, in the domain of “mitigating the spread of Covid-19,” mask wearing would be quite ordinary, whereas volunteering for an experimental trial of an untested vaccine or aggressively confronting people who do not comply with mitigation guidelines would be more extreme. Extreme actions differ from moderate actions in how unusual they are and the costs they impose on people who engage in them (*11*). Many extreme behaviors are also socially undesirable, although there are exceptions (*12*). It is important to understand the determinants of extreme actions, especially in the political domain, because such actions can cause material and psychological harm to individuals, and collectively, they can threaten the stability of democratic governance (*13*).

The current work proposes that attitudinal ambivalence can increase support for extreme political action despite research from a variety of contexts indicating that greater ambivalence typically dampens attitude-consistent behavior [e.g., (*14*–*18*)]. For example, people are typically less likely to vote in an election or for a candidate about which they feel conflicted (*7*–*9*). Traditionally, therefore, attitudinal ambivalence has been conceptualized as an indication of a “weak” attitude (*19*). Our prediction, though, is that the political behavioral implications of ambivalence reverse as a function of behavioral extremity—that is, ambivalence simultaneously increases the tendency to engage in extreme attitude-consistent political behavior as it dampens engagement in moderate action.

Extreme political behaviors are not usually appealing, but they can become more attractive when a person’s political attitudes come under psychological threat, motivating them to produce some sort of defensive response (*10*, *20*–*22*). The appeal of extreme behaviors as defensive responses to attitudinal threats might be due to their unusualness and how costly they are, which make them seem diagnostic of individuals’ personal beliefs (*11*, *23*–*24*). That is, extreme actions send strong signals about the commitments of the people who perform them. We suggest that being ambivalent can be quite threatening and uncomfortable (*25*–*26*), especially regarding issues that matter a lot to the person, and like other people experiencing attitude-relevant psychological threats (*10*, *20*–*22*), ambivalent people are drawn to the clarity and commitment that extreme behaviors signal.

Although our interaction hypothesis about ambivalence has not been explored previously, there is some evidence that ambivalence-induced discomfort can motivate an extreme response. For example, previous research on “ambivalence amplification” in the domain of prejudice shows that when people hold unfavorable attitudes toward an outgroup but want to be egalitarian (i.e., their outgroup attitudes are ambivalent), attempts to distance themselves from their prejudiced reactions can result in evaluating and treating individual outgroup members with exaggerated favorability and approach-oriented behaviors [e.g., (*27*–*29*)]. This could reflect a desire to be less ambivalent about the outgroup (*30*–*31*) or at least to ensure that one’s ambivalence does not have undesired effects on judgments about or behavior toward the outgroup. Our proposed ambivalence-extremity relationship might similarly involve trying to reduce or mitigate the effects of ambivalence by being willing to engage in extreme action in accord with one’s position.

However, people do not always try to counteract their ambivalence, suggesting another explanation. At times, people accept their ambivalence because they believe that this accurately reflects the underlying information (*32*–*33*). Feeling justifiably ambivalent in this way might still be uncomfortable, especially for those with strong opinions, which could make extreme behaviors appealing. However, if ambivalence increases extreme behaviors when people believe it is justified, this would indicate that the effect does not necessarily involve an attempt to reduce the ambivalence. Rather, this result would be more consistent with a different kind of defensive response that has been documented in the literature, namely, “symbolic self-completion” (*34*). Symbolic self-completion involves attempting to look or feel stronger in a domain where one feels weak. For example, receiving negative (versus positive) feedback about one’s potential in a self-definitional domain (e.g., career) can motivate a person to look for ways to signal competence in that domain, even if doing so comes at the expense of other salient goals (*35*). The colloquial term for this type of behavior—“overcompensating”—highlights that symbolic self-completion can often appear over-the-top to other people. People are more likely to resort to this when they cannot directly address the source of their discomfort (*34*), such as if they believe their ambivalence is appropriate. The proposed relationship could therefore result from ambivalent people being attracted to the signal value associated with extreme behaviors, which enable them to appear to themselves and others as if they held clearer and more decisive positions than they actually do.

Both explanations are compatible with ambivalence-induced discomfort increasing extreme actions, but they suggest different conditions under which this should occur, and different interventions that should reduce it. On the one hand, if ambivalence increases extreme actions due to an attempt to reduce the ambivalence, then persuading people that ambivalence is justified and appropriate should prevent the effect from emerging by undercutting its motivational impetus (i.e., by making ambivalence less threatening and ambivalent people more comfortable with their position). On the other hand, if the effect results from people who feel justifiably ambivalent trying to signal strength to compensate for weakness, then telling them that ambivalence is justified would merely reinforce what they already believe and would not reduce the effect. Rather, in this case, persuading people that ambivalence is unjustified and inappropriate might be more likely to prevent the effect’s emergence by making ambivalent people doubt the validity of their position. After documenting the ambivalence-extremity link, we present a critical experimental test to tease these alternative explanations apart.

Consistent with standard practices in attitudinal ambivalence research, we conceptualize and operationalize ambivalence in terms of both the subjective experience of feeling conflicted about one’s attitude and the objective simultaneous presence of both positive and negative evaluations of the attitude object (*3*, *5*). When measured in relation to the same attitude object, these “subjective” and “objective” approaches to measuring ambivalence are typically moderately-to-strongly correlated (*4*) and they often have similar effects [but not always, e.g., (*36*)]. However, we theorized that subjective ambivalence (felt conflict) might play a stronger role here due to its clearer link to psychological discomfort (*25*–*26*) relative to objective measures (*37*). As such, although we also examined objective ambivalence in some studies, we emphasize subjective ambivalence empirically and in our discussion.

Altogether, we propose that ambivalence-induced discomfort makes supporting extreme forms of pro-attitudinal political action more appealing. Therefore, support for extreme pro-attitudinal action increases as ambivalence increases. These predictions are grounded in research on ambivalence-induced discomfort (*25*–*29*) and are broadly consistent with perspectives on how people cope with and compensate for psychological threats (*10*, *20*–*22*). However, they contrast starkly with extensive previous research examining how attitudinal ambivalence decreases engagement in more ordinary forms of attitude-consistent behavior (*7*–*9*, *14*–*18*). In essence, we suggest that its association with discomfort can make ambivalence a double-sided attitude property with respect to the attitude’s “strength” [i.e., its impact on behavior; (*11*, *19*, *38*–*40*)]. We sought to demonstrate this by showing that greater attitudinal ambivalence both decreases and increases support for pro-attitudinal behavior, depending on the behavior’s extremity.

## RESULTS

We tested our predictions in six studies. In study 1, we obtained an initial test of our hypotheses in two publicly available national survey datasets (combined *N* = 6538). We examined associations between partisan ambivalence and support for partisan violence (an extreme pro-attitudinal form of political action) as well as voting intentions (an ordinary form of political action), with and without relevant statistical controls. In study 2 (*N* = 5082), we meta-analyzed all the relevant data collected in our laboratory over approximately a 3-year period in which we measured ambivalence about one’s position on one of several different sociopolitical topics and willingness to engage in various extreme and often also moderate behaviors in support of one’s position on that same topic. We recruited a new sample to provide extremity ratings of the behaviors and merged these ratings with our main dataset to test behavioral extremity as a moderator of the behavioral implications of ambivalence. In studies 1 and 2, we also tested robustness by estimating and statistically controlling for response biases and other covariates. In study 3 (*N* = 244), we investigated the specificity of the effect of political ambivalence to extreme political versus nonpolitical behavior, addressing potential alternative explanations by examining an implication of our theorizing (i.e., the effect should be attitude-specific, occurring within-domain). In study 4 (*N* = 374), we examined feelings of attitudinal discomfort—an established consequence of ambivalence—as a mediator of the effect of ambivalence on support for partisan violence. In study 5 (*N* = 250), we replicated and extended the previous studies using a measure of committing to donate real money (i.e., potential raffle winnings) to real environmental organizations known for using extreme tactics. Finally, in study 6 (*N* = 567), we experimentally tested whether the effect occurs because ambivalent people try to reduce their ambivalence by supporting extreme actions, or alternatively, whether the discomfort associated with being ambivalent increases support for extreme actions even (or especially) when people believe that ambivalence is justified.

### Study 1—Partisan ambivalence predicting support for partisan violence and voting intentions in national survey data

In study 1, we sought initial evidence for our hypotheses by analyzing two publicly available national surveys: the Nov-Dec 2019 Views of the Electorate Research Survey (VOTER; *N* = 5622) and the LSU team module of the 2020 Cooperative Election Study (CES; *N* = 916). Each included the measures needed to compute the same established (objective) measure of partisan ambivalence [e.g., (*7*–*8*)] using the standard similarity-intensity formula [e.g., (*3*–*5*, *41*)] as well as a set of four commonly used measures of support for partisan violence (SPV) [e.g., (*42*–*44*)]. As noted, although our theorizing centers on subjective ambivalence (felt conflict) because of its more direct implications for discomfort and defensive motivation, subjective and objective ambivalence are often moderately-to-strongly correlated and often produce similar effects (*4*), so we thought that the phenomenon could also emerge using this measure, despite offering a more conservative test. The SPV measures are well suited for testing our hypotheses because they are specified to be pro-attitudinal (i.e., to support respondents’ own political side, not the opposing side), which our framework requires. Intentions to vote in 2020 were also assessed in each survey, allowing us to examine both extreme and moderate behaviors. Finally, we also examined age, gender, education, political interest, political ideology, and several other known or potential psychological predictors of SPV as covariates.

**Fig. 1.**
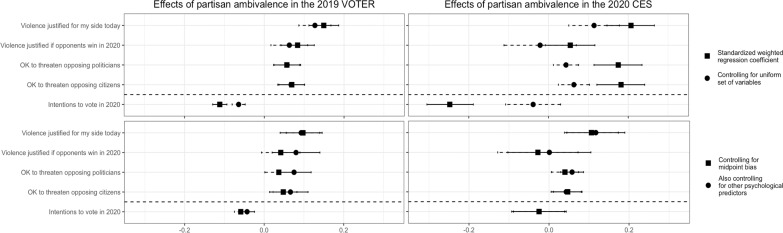
Partisan ambivalence and support for partisan violence. Associations between partisan ambivalence and SPV (above dashed line) as well as voting intentions (below dashed line) beyond the effects of key statistical controls. Error bars are 95% CIs.

We tested the effect of partisan ambivalence on SPV under different model specifications. We regressed each SPV measure onto ambivalence alone (first analyses), then added controls for a uniform set of covariates (second analyses) and response biases (third analyses), and finally added additional predictors of SPV (fourth analyses). Results are presented in Fig. 1. Consistent with expectations, small but significant positive effects of ambivalence emerged for all four SPV measures in VOTER and three in CES while simultaneously negatively predicting voting intentions, and these conclusions held when standard covariates (age, gender, education, political interest, political ideology) were included. It was particularly important to control for political interest and ideology as both have been linked to attitudinal ambivalence in previous work (*45*). Separate analyses of Democrats and Republicans showed that the effect generally emerged among both parties, with the first, most face valid SPV item (how justified respondents thought it currently was for their party to use violence to advance their political goals) producing the strongest and most consistent effect across both parties. To further test robustness, we then constructed a measure to assess whether the pattern of results could be due to a bias to provide non-extreme (i.e., near-midpoint) responses, which could be related to ambivalence (*46*), and controlled for it in the third set of analyses. Doing so attenuated the VOTER effects slightly, but three remained significant, and the CES results seemed unaffected. We also constructed a measure of acquiescence bias, i.e., the tendency to provide high responses across measures, and controlled for it instead of and in addition to midpoint bias, which also did not change the conclusions. We then added further variables to the model that have predicted SPV previously [CES: strength of partisan identification, trait aggression, (*43*); VOTER: perceptions of whether the out-party has good/bad intentions, (*44*)] or could plausibly do so [CES: political knowledge; e.g., (*47*)]. As shown in Fig. 1, the same conclusions largely held. These effects of partisan ambivalence on SPV were also robust to controlling for the in-party feeling thermometer (a component of the partisan ambivalence measure), and the in-party thermometer did not significantly positively predict any SPV item in either sample.

The predicted effect thus emerged in national survey data on Americans’ SPV. It also proved robust to key statistical controls, including response biases and other relevant psychological and demographic variables, although the effects were small with these covariates taken into account. As mentioned, this measure of partisan ambivalence is just a proxy for the construct we theorized is directly influential—felt attitudinal conflict. So, having obtained these initial results for ambivalence, we proceeded to measure felt conflict in our subsequent studies.

### Study 2—Meta-analysis of ambivalence predicting behavioral willingness self-report measures varying in extremity

In study 2, we sought to conceptually replicate the effects of ambivalence on behavioral extremity across a range of behaviors and when using a subjective (versus objective) ambivalence measure. To do so, we compiled all the data we had previously collected over 3 years of research on related topics in which we had measured political attitudinal ambivalence and willingness to engage in various moderate (e.g., voting) and extreme (e.g., engaging in violence) pro-attitudinal behaviors, often while testing other hypotheses about attitude strength [i.e., attitude polarization and certainty; (*38*)] and behavioral intentions. We did not exclude any datasets containing measures of political attitudinal ambivalence and willingness to engage in extreme behaviors. In total, we had 19 studies with a subjective measure of political attitudinal ambivalence and at least one extreme behavioral willingness measure (*N* = 5082). Seven of the samples also contained an objective ambivalence measure (*N* = 1702). We did not have any datasets with objective but not subjective ambivalence.

Participants reported their subjective ambivalence [i.e., how mixed/conflicted/indecisive they felt; (*4*)] and sometimes their objective ambivalence [how much they reported both positive and negative evaluations of their position; (*3*)], about either their political ideology in general or their position on a specific political issue—COVID mitigation policies, abortion, gun control, national identity, or racial/ethnic identity {objective-subjective correlation aggregated across topics: *r*(1700) = 0.70, *P* < 0.0001, 95% confidence interval (CI): [0.67, 0.72]}. Participants reported their willingness to engage in various behaviors in support of their ideology or position on the specific issue they had been asked about. That is, the ambivalence and behavioral willingness measures in each study always referred to the same topic (e.g., ambivalence about COVID-19 mitigation and willingness to engage in COVID-19 mitigation–related behaviors). The specific behaviors measured varied across studies, as summarized in Table 1 and broken down fully in table S1. We measured ambivalence before the extreme behavioral willingness measures in 13 samples and afterward in 6, and the same relationship between them generally held regardless of order, as shown in the Supplementary Materials.

**Table 1.** Attitude-consistent behaviors varying in extremity. Presented from most to least extreme. Mean behavioral willingness, number of samples (*k*), and combined sample size (*N*) are also presented. Measures with * were also assessed in study 6.

Behavior	Extremity	Willingness	Samples and sample size
*Sacrifice life: One of two scales depending on sample (see Materials and Methods)	6.64	2.69	*k* = 7, *N* = 1489
*Commit violence: *To what extent would you be willing to engage in violence on behalf of your* … either *political beliefs and opinions?* or *position on mask wearing requirements?* or *position on taking recommended COVID-19 precautions?*	6.58	1.67	*k* = 12, *N* = 3219
*Fight opponents: *If someone was behaving in a way that is opposed to your views about [X], to what extent would you be willing to fight them?*	5.88	2.36	*k* = 19, *N* = 4909
*Confront opponents: *To what extent would you be willing to aggressively confront someone who was acting in a way that is opposed to your* … either *political approach?* or *views about mask wearing requirements?*	5.80	2.55	*k* = 6, *N* = 1501
*Get opponents fired: *To what extent would you be willing to try to get someone who disagrees with you about mask wearing requirements fired from their job?* or *If someone was behaving in a way that is opposed to your political beliefs and opinions, to what extent would you be willing to try to get them fired from their job?*	5.24	2.30	*k* = 3, *N* = 914
COVID vaccine trial: *To what extent are you willing to participate in an experimental clinical trial of a previously untested vaccine against COVID-19?*	5.20	3.55	*k* = 2, *N* = 634
COVID treatment trial: *If you became sick with COVID-19, to what extent would you be willing to participate in an experimental clinical trial of a new and previously untested treatment for the illness?*	5.13	4.36	*k* = 2, *N* = 634
Self-isolation (COVID): *To what extent are you willing to completely isolate yourself*…*to help prevent the spread of COVID-19?* (where… is either *from all other people you do not live with until the pandemic is over* or *for the next year from all other people you do not live with*)	5.03	3.95	*k* = 5, *N* = 1375
*Block opponents: *To what extent would you be willing to block on all social media and discontinue all contact with someone who disagrees with you about mask wearing requirements?*	5.03	3.40	*k* = 1, *N* = 402
*Argue with opponents: *To what extent would you be willing to get into a heated argument with someone if they attacked your political beliefs and opinions?*	4.86	2.85	*k* = 4, *N* = 979
Make sacrifices: *To what extent would you be willing to make sacrifices on behalf of your political beliefs and opinions?*	4.08	4.02	*k* = 2, *N* = 560
Change opponents’ behavior: *If someone was behaving in a way that is opposed to your views about [X], to what extent would you be willing to try to change their mind?*	3.92	4.63	*k* = 2, *N* = 702
*Display views: *To what extent would you be willing to publicly advertise your political beliefs and opinions (e.g., via social media, a bumper sticker, t-shirt, etc.)?*	3.26	3.57	*k* = 3, *N* = 715
*Advocate: *To what extent would you be willing to advocate to others on behalf of your political beliefs and opinions?* or *To what extent would you be willing to advocate for your political approach?* or *To what extent are you willing to try to persuade others who disagree with you about mask wearing requirements?*	3.06	4.69	*k* = 9, *N* = 2438
Avoid crowds (COVID): *To what extent are you willing to avoid… to help prevent the spread of COVID-19?* (where… was either *large crowds of people* or *crowds of 50 or more people for the next month*)	2.79	5.62	*k* = 6, *N* = 1485
*Donate money: *To what extent would you be willing to donate money to causes that support your political beliefs and opinions?*	2.72	3.96	*k* = 6, *N* = 1623
Get vaccinated (COVID): *To what extent are you willing to get vaccinated against COVID-19 when the vaccine becomes available?*	2.72	5.52	*k* = 2, *N* = 634
*Volunteer: *To what extent would you be willing to volunteer your time to support causes that agree with your political approach?*	2.71	4.46	*k* = 1, *N* = 300
*Put in effort: *To what extent would you be willing to put effort into supporting your political beliefs and opinions?*	2.50	4.71	*k* = 2, *N* = 560
Wear mask (COVID): *To what extent are you willing to wear a mask in public to help prevent the spread of COVID-19?*	2.46	6.37	*k* = 5, *N* = 1201
Spend time: *To what extent would you be willing to spend time supporting your political beliefs and opinions?*	2.41	4.54	*k* = 2, *N* = 560
Get treated (COVID): *If you became sick with COVID-19, to what extent would you be willing to take the first newly developed and approved medications for the illness?*	2.41	4.94	*k* = 2, *N* = 634
*Political support: *To what extent are you willing to support political candidates who share your views on mask wearing requirements?*	2.41	5.27	*k* = 2, *N* = 611
Follow COVID mitigation guidelines: *To what extent would you be willing to follow recommendations regarding health precautions during the COVID-19 pandemic?*	2.32	6.55	*k* = 1, *N* = 110
Wash/sanitize hands (COVID): *To what extent would you be willing to wash or sanitize your hands regularly when outside your home to help prevent the spread of COVID-19?*	1.97	6.68	*k* = 1, *N* = 110
*Vote: *To what extent would you be willing to vote for political candidates who agree with your political beliefs and opinions*?	1.92	6.11	*k* = 7, *N* = 1827
*Read news: *To what extent would you be willing to read newspaper articles that support your political beliefs and opinions?*	1.73	5.35	*k* = 1, *N* = 252

#### Normative behavioral extremity ratings

We recruited a separate sample to rate how extreme engaging in the behaviors we assessed would be (1 = Not at all, 7 = Very much; 69 < *n* < 72, depending on behavior; see Table 1). We expected the extremity ratings to moderate the effect of both objective and subjective ambivalence on behavioral willingness.

#### Behavioral extremity as a categorical moderator

We constructed extreme and moderate behavioral willingness composite measures by calculating the mean within each sample of variables that had extremity ratings significantly above or below the midpoint at the *P* < 0.001 level (which all but two behaviors did; see Table 1). With these composites, we conducted a mixed regression analysis with ambivalence (continuous, between-subjects), behavioral extremity (categorical, within-subjects), and their interaction predicting behavioral willingness. Averaging across a variety of measures is appropriate for an initial analysis because in this approach, both the ambivalence and behavioral willingness measures are at the same level of generality [i.e., both capture the person’s general position on the issue; (*48*)]. Examining subjective ambivalence first, we found a significant interaction with behavioral extremity, β = 0.65, SE = 0.03, *t*_7926.55_ = 20.19, *P* < 0.0001. Examining extreme versus moderate behaviors separately, whereas subjective ambivalence positively predicted extreme behavioral willingness, β = 0.45, SE = 0.01, *t*_4970_ = 20.84, *P* < 0.0001, 95% CI: [0.40, 0.49], it negatively predicted moderate behavioral willingness, β = −0.29, SE = 0.01, *t*_3396_ = −12.17, *P* < 0.0001, 95% CI: [−0.34, −0.25]. Replacing subjective with objective ambivalence replicated this result {interaction: β = 0.72, SE = 0.08, *t*_2074.62_ = 9.32, *P* < 0.0001; extreme: β = 0.55, SE = 0.04, *t*_1700_ = 13.18, *P* < 0.0001, 95% CI: [0.47, 0.63]; moderate: β = −0.23, SE = 0.04, *t*_682_ = −5.17 *P* < 0.0001, 95% CI: [−0.31, −0.14]}.

#### Behavioral extremity as a continuous moderator

Having obtained support for our prediction at the most general level, we next examined behavioral extremity as a continuous variable, using the extremity ratings from the independent sample to predict the direction and magnitude of the ambivalence-willingness correlations. This more granular analysis enables us to determine whether ambivalence-willingness relations become less negative and then increasingly positive as behavioral extremity increases. For subjective ambivalence, we meta-analyzed the results across samples by conducting a multilevel model. In contrast to “two-step” approaches that model aggregate data from each study, this “one-step” meta-analysis approach models individual participant data from all studies (*49*), allowing for greater flexibility in model specification, such as the inclusion of covariates and the ability to account for variance associated with the topics used. This meta-analysis provides an unbiased effect size estimate by including every data point collected in this line of work, consistent with recent recommendations for best research practices (*50*–*52*).

We specified a three-level model predicting behavioral willingness in which we nested willingness ratings (level 1) within topics (level 2) within the type of behavior (level 3). We nested within topics rather than studies as multiple studies used the same topics, and we thought that it was more important to account for variance associated with topic than study. We entered the behavioral extremity ratings (grand-mean centered) at level 3 and subjective ambivalence (group-mean centered within behavior type) at level 2, along with their interaction. We specified random slopes and intercepts for all predictors. The interaction between ambivalence and behavioral extremity was significant, β = 0.36, SE = 0.06, *t*_25.88_ = 6.48, *P* < 0.0001, indicating that the effect of ambivalence on behavioral willingness depended on behavioral extremity. Simple effects analyses confirmed that the effect of ambivalence was positive when behavioral extremity was 1 SD above its mean, β = 0.51, SE = 0.09, *t*_19.60_ = 5.96, *P* < 0.0001, 95% CI: [0.33, 0.69], and negative when extremity was 1 SD below its mean, β = −0.21, SE = 0.07, *t*_36.31_ = −3.01, *P* = 0.005, 95% CI: [−0.35, −0.07]. The objective measure of ambivalence also replicated these effects {interaction: β = 0.44, SE = 0.08, *t*_14.25_ = 5.54, *P* < 0.0001; simple effect, high extremity: β = 0.64, SE = 0.10, *t*_11.18_ = 6.27, *P* < 0.0001, 95% CI: [0.42, 0.87]; simple effect, low extremity: β = −0.25, SE = 0.12, *t*_21.21_ = −2.23, *P* = 0.037, 95% CI: [−0.48, −0.02]}.

#### Robustness checks

Beyond these comprehensive analyses, we also conducted a more focused analysis, excluding a subset of our data about which reviewers had some validity concerns: two moderate behavioral willingness measures with small samples [washing hands during COVID; ambivalence correlation: *r*(108) = −0.47; following COVID mitigation guidelines: *r*(108) = −0.46] and one extreme behavioral willingness measure that seems less clearly pro-attitudinal than the others [sacrificing one’s life for the vaccine or economy: *r*(789) = 0.68]. We still had four samples with a more straightforward measure of willingness to sacrifice one’s life. Although the excluded variables support the same directional conclusions, there could be some reservations about placing much weight on them in interpreting the results, especially with respect to effect sizes. The focused analysis is intended to increase confidence in our effect size estimates with the more questionable measures removed.

**Fig. 2.**
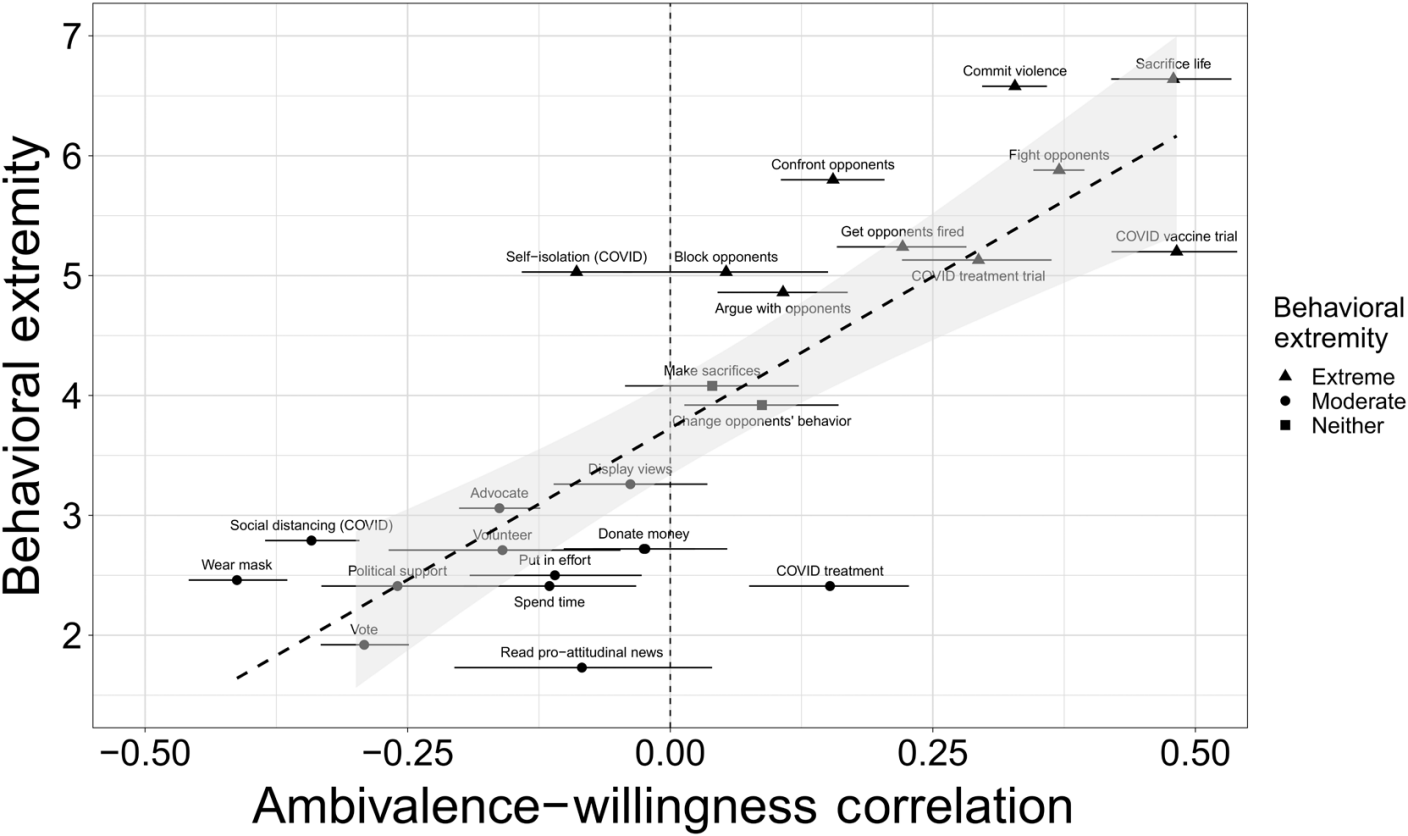
Subjective ambivalence correlations with behavioral willingness as a function of behavioral extremity. Error bars are 95% CIs.

Re-running our multilevel model after excluding these three behavioral willingness measures replicated the same conclusions as with the full dataset: a significant interaction between subjective ambivalence and behavioral extremity [β = 0.32, SE = 0.06, *t*_24.59_ = 5.70, *P* < 0.0001] with a positive effect at high extremity {β = 0.45, SE = 0.08, *t*_19.08_ = 5.32, *P* < 0.0001, 95% CI: [0.27, 0.62]} and a negative effect at low extremity {β = −0.19, SE = 0.07, *t*_33.70_ = −2.63, *P* = 0.013, 95% CI: [−0.34, −0.04]}. The objective measure of ambivalence also again replicated these results. Figure 2 makes the consistency of the reversal apparent—only one negative association emerged between ambivalence and an extreme behavior (self-isolation during COVID-19), which might be an artifact of measurement timing: self-isolation likely seemed less extreme when we measured willingness to do it in mid-2020 (when many people were self-isolating) than when we measured how extreme it seemed in mid-2022. Similarly, on the moderate side, there was only one positive ambivalence-willingness correlation (getting treated for COVID-19), which might have seemed more extreme in 2020 (when such treatments were less established) than in 2022. Despite the difference in measurement timing likely adding some noise to the data, the effect still emerged. Table S1 presents the correlations for each measure within each sample separately.

**Fig. 3.**
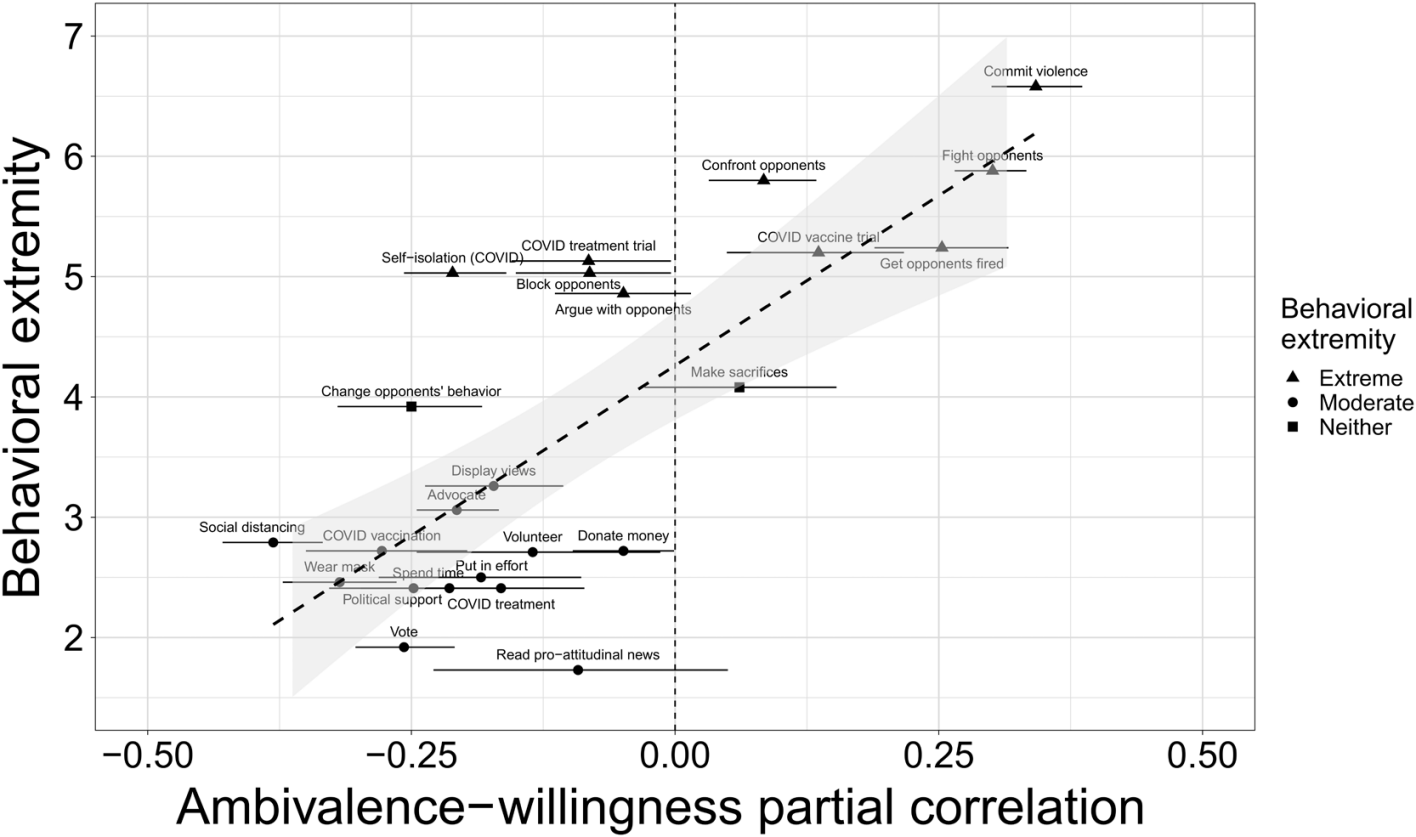
Subjective ambivalence partial correlations with behavioral willingness as a function of behavioral extremity. Controlling for midpoint and acquiescence response biases. Error bars are 95% CIs.

Our findings were also robust to controlling for midpoint and acquiescence response biases (detailed in Materials and Methods) and their interactions with behavioral extremity {ambivalence × behavioral extremity interaction: β = 0.20, SE = 0.03, *t*_21.89_ = 5.91, *P* < 0.0001; simple effect of ambivalence, high extremity: β = 0.16, SE = 0.05, *t*_15.80_ = 3.08, *P* = 0.007, 95% CI: [0.05, 0.27]; simple effect, low extremity: β = −0.25, SE = 0.04, *t*_32.67_ = −5.86, *P* < 0.0001, 95% CI: [−0.33, −0.16]}. Partial correlations with midpoint and acquiescence biases controlled, shown in Fig. 3, illustrate this. (Note that these analyses exclude six samples in which we could not control for response biases due to insufficient measures.) The same conclusions also held when further controlling for attitude certainty (*53*) and its interaction with behavioral extremity, available in all 19 samples [ambivalence × extremity interaction: β = 0.17, SE = 0.04, *t*_21.61_ = 4.73, *P* = 0.0001], or political ideology (*45*, *54*) and its interaction with extremity, available in 11 samples [ambivalence × behavioral extremity interaction: β = 0.14, SE = 0.05, *t*_8.76_ = 2.63, *P* = 0.028], or for any combination of covariates and their interactions with behavioral extremity.

#### Attitude polarization as a moderator

We next assessed potential moderation by attitude polarization (i.e., the reported attitude’s distance from the scale neutral midpoint), for two reasons. First, our framework assumes that feeling conflicted about a motivationally potent attitude produces defensiveness that makes extreme behaviors more appealing. Thus, identifying participants for whom the attitude is especially motivating, such as those with polarized attitudes (*55*–*56*), should enhance the effect. That is, the need to justify one’s view in light of being ambivalent should be magnified the more extreme one’s position is. Second, research on attitudinal ambivalence frequently examines ambivalence as a moderator of the effect of attitude polarization on attitude-consistent behavior [e.g., (*14*, *57*)], so our methodology here mirrors that well-established approach. However, that previous literature has focused on how ambivalence moderates the attitude-behavior relationship for ordinary behaviors, whereas we flip this, examining how attitudes moderate the ambivalence-behavior relationship for extreme behaviors too.

Note that whereas most of our samples had participants report their ambivalence about an attitude measure, seven samples instead had participants report their ambivalence about a political ideology measure and five additional samples included a separate political ideology measure (although ambivalence in those studies referred to a traditional attitude measure). We were unsure whether ideological polarization should moderate the effect of ambivalence in the same manner that we expect for attitude polarization, although it seemed plausible that it might do so. We therefore report analyses of moderation by ideological (versus attitude) polarization separately, on a more exploratory basis.

We used the categorical measures of extreme and moderate behavioral willingness for simplicity. A multiple regression testing the effects of ambivalence, attitude polarization, and behavioral extremity found that the three-way interaction was not significant, β = −0.02, SE = 0.04, *t*_4559.19_ = −0.55, *P* = 0.583. However, for extreme behavioral willingness, a significant ambivalence × polarization interaction emerged, β = 0.09, SE = 0.03, *t*_3143_ = 3.09, *P* = 0.002. Whereas ambivalence had a positive effect even when the attitude was low in polarization (−1 SD), β = 0.47, SE = 0.04, *t*_3143_ = 11.73, *P* < 0.0001, 95% CI: [0.38, 0.56], this effect was stronger when the attitude was high in polarization (+1 SD), β = 0.64, SE = 0.04, *t*_3143_ = 15.84, *P* < 0.0001, 95% CI: [0.56, 0.72]. The two-way interaction was not significant for moderate behavioral willingness, β = 0.05, SE = 0.03, *t*_1564_ = 1.83, *P* = 0.067, 95% CI: [0.03, 0.16]. Here, ambivalence had a negative effect both when attitude polarization was low, β = −0.20, *t*_1564_ = −4.53, *P* < 0.0001, 95% CI: [−0.29, −0.12], and when it was high, β = −0.10, *t*_1564_ = −2.31, *P* = 0.021, 95% CI: [−0.18, −0.02].

Objective ambivalence did not significantly interact with attitude polarization in predicting extreme behavioral willingness, β = 0.08, SE = 0.04, *t*_1698_ = 1.80, *P* = 0.071. It instead positively predicted extreme behaviors at low and high attitude polarization (βs > 0.60, *t*s > 8.6, *P*s < 0.0001). Objective ambivalence and attitude polarization did significantly interact in predicting moderate behavioral willingness, β = 0.18, SE = 0.05, *t*_680_ = 3.75, *P* = 0.0002, such that ambivalence had a significant negative effect when polarization was low, β = −0.26, SE = 0.08, *t*_680_ = −3.36, *P* = 0.0008, 95% CI: [−0.40, −0.10], but not high, β = 0.11, SE = 0.06, *t*_680_ = 1.88, *P* = 0.060, 95% CI: [−0.00, 0.22]. The three-way interaction between objective ambivalence, attitude polarization, and behavioral extremity also did not reach significance, β = −0.14, SE = 0.08, *t*_2093.55_ = −1.84, *P* = 0.066.

Broadly similar conclusions emerge from analyzing ideological polarization as a moderator in place of attitude polarization. Specifically, a significant two-way interaction emerged between subjective ambivalence and ideological polarization in predicting extreme behaviors, β = 0.11, SE = 0.02, *t*_3215_ = 4.59, *P* < 0.0001. Although ambivalence had a positive effect even among ideological moderates, β = 0.22, SE = 0.03, *t*_3215_ = 6.51, *P* < 0.0001, 95% CI: [0.15, 0.28], its effect was stronger among ideological extremists, β = 0.43, SE = 0.03, *t*_3215_ = 13.21, *P* < 0.0001, 95% CI: [0.37, 0.49]. The two-way interaction between objective ambivalence and ideological polarization was also significant, β = 0.32, SE = 0.07, *t*_398_ = 4.59, *P* < 0.0001, with a significant effect of ambivalence among ideological extremists, β = 0.58, SE = 0.09, *t*_398_ = 6.54, *P* < 0.0001, 95% CI: [0.41, 0.75], but not among ideological moderates, β = −0.07, SE = 0.10, *t*_398_ = −0.69, *P* = 0.490, 95% CI: [−0.26, 0.12]. For moderate behaviors, ideological polarization moderated the effect of both subjective ambivalence [β = 0.08, SE = 0.02, *t*_3110_ = 3.35, *P* = 0.0008] and objective ambivalence [β = 0.16, SE = 0.06, *t*_398_ = 2.58, *P* = 0.010] such that the negative effect of ambivalence was stronger among ideological moderates than extremists.

#### Summary of study 2 results

In sum, behavioral extremity moderated the association between (subjective and objective) attitudinal ambivalence and willingness to engage in pro-attitudinal forms of political action. Whereas the typical negative relationship emerged for moderate behavior, this reversed for extreme behaviors. As described, this was robust to controlling statistically for indices of response bias as well as attitude certainty and political ideology. Furthermore, the reversal for extreme behaviors was enhanced by greater attitude (or ideological) polarization, consistent with magnification of the effect of ambivalence on extreme behavior when the attitude is especially motivating for the person.

### Study 3—Within-domain versus across-domain effects

We conducted study 3 (*N* = 244) to further address response bias as an alternative explanation for the effect, using two different methods. First, we examined whether the effects of ambivalence and behavioral extremity would be limited to behavioral measures within the same domain (i.e., political ambivalence and political behaviors) as our framework predicts, versus extending to irrelevant domains, which we do not predict but could indicate response bias. Second, we examined whether individual differences in the tendency toward thoughtfulness and/or reflectiveness, operationalized as the need for cognition (*58*) and the cognitive reflection test (*59*), might explain the effect. Such tendencies would not be involved in the process we propose is occurring, but they might be related to ambivalence and could then plausibly drive response patterns consistent with our data.

Our argument is that ambivalence-induced discomfort makes supporting extreme pro-attitudinal behaviors more appealing. For a behavior to be pro-attitudinal, it logically must occur within the same domain as the ambivalent attitude [“within-domain compensation;” e.g., (*60*)] whereas behaviors in domains that are unrelated to the ambivalent political attitude should not be affected by the ambivalence-induced discomfort. Thus, we expected greater subjective political attitudinal ambivalence to be associated with more support for extreme pro-attitudinal political action, but not extreme behavior in support of one’s position on an unrelated topic. To examine this, we assessed willingness to engage in extreme and moderate behaviors in support of one’s (i) political views and (ii) romantic relationship (eligibility for the study was limited to those in a relationship). Specifically, we measured two extreme and two moderate behaviors in each domain (eight total). The measures were framed identically across domains except that they were described as supporting either the person’s political beliefs or their romantic relationship. The two extreme behaviors were (i) aggressively confronting someone who was attacking one’s [political beliefs/romantic relationship] and (ii) committing violence to defend one’s [political beliefs/romantic relationship], and the two moderate behaviors were (i) volunteering an hour of one’s time to benefit one’s [political beliefs/romantic relationship] and (ii) spending a modest sum of money to benefit one’s [political beliefs/romantic relationship]. The behaviors correlated highly only within extremity and domain categories, so we averaged the two respective items to create four composite variables—extreme political, moderate political, extreme relationship, and moderate relationship behaviors.

We examined the associations of the behavioral willingness measures with political ambivalence by conducting mixed regression analyses using the MEMORE macro in SPSS (*61*), with subjective political ambivalence (continuous, between-subjects), behavioral extremity (categorical, within-subjects), and their interaction predicting willingness to engage in political behaviors. We then repeated this analysis with the relationship behavior measures. The results were consistent with our account and inconsistent with response bias. First, we replicated the findings from studies 1 and 2: Behavioral extremity significantly moderated the effect of ambivalence on political behavioral willingness, β = 0.32, SE = 0.11, *t*_242_ = 3.00, *P* = 0.003, such that it was positive and significant for extreme behaviors, β = 0.16, SE = 0.08, *t*_242_ = 2.07, *P* = 0.040, 95% CI: [0.01, 0.31], and negative (albeit nonsignificant) for moderate behaviors, β = −0.16, SE = 0.11, *t*_242_ = −1.46, *P* = 0.147, 95% CI: [−0.38, 0.06]. In contrast, ambivalence was not related to willingness to engage in extreme relationship-focused behaviors, β = −0.04, SE = 0.13, *t*_242_ = −0.31, *P* = 0.755, 95% CI: [−0.29, 0.21], or moderate ones, β = −0.09, SE = 0.09, *t*_242_ = −1.04, *P* = 0.299, 95% CI: [−0.26, 0.08] [and these associations did not differ, β = 0.05, SE = 0.13, *t*_242_ = 0.38, *P* = 0.701].

We then examined whether the effects on political behavioral willingness hold beyond any effects of need for cognition and cognitive reflection. First, contrary to the idea that ambivalence produces divergent effects on extreme versus moderate behavioral willingness because it is associated with being more thoughtful or reflective, we found that ambivalence was negatively correlated with need for cognition, *r*(242) = −0.21, *P* = 0.001, and not significantly correlated with cognitive reflection, *r*(242) = −0.06, *P* = 0.382. Furthermore, controlling for these variables left the association between ambivalence and extreme behavioral willingness unchanged, *r*(240) = 0.14, *P* = 0.030, although it directionally attenuated the association between ambivalence and moderate behavioral willingness, *r*(240) = −0.05, *P* = 0.441. Overall, then, we found that behavioral extremity only moderated the effect of ambivalence on behavioral willingness within the same domain, and the positive relationship between ambivalence and extreme behavioral willingness was unrelated to individual differences in thoughtfulness or cognitive reflection.

### Study 4—Mediation through attitudinal discomfort

We conducted study 4 to obtain evidence for our proposed mechanism, attitudinal discomfort. Increased attitudinal discomfort at higher levels of ambivalence would conceptually replicate much previous research showing that ambivalence is associated with unpleasant feelings stemming from cognitive inconsistency (*25*–*27*). Furthermore, our framework predicts that the discomfort associated with ambivalence should mediate the effect of ambivalence on support for partisan violence.

We assessed whether the effect of subjective ambivalence on support for partisan violence was mediated by attitudinal discomfort. A Mechanical Turk sample (*N =* 374) completed the subjective ambivalence measure from studies 2 and 3 (referring to their “political views”) and the study 1 SPV measures and reported how uncomfortable they felt about their ideological position (“How uncomfortable are you with your ideological views?”). Subjective ambivalence and discomfort were related, as expected [*r*(372) = 0.55, *P* < 0.001]. Echoing the study 1 results, ambivalence significantly predicted the first, most face valid SPV measure (i.e., support for violence today) {β = 0.13, SE = 0.06, *t*_372_ = 2.04, *P* = 0.042, 95% CI: [0.01, 0.25]}, and the indirect effect through discomfort was significant (β = 0.12, SE = 0.04, 95% CI: [0.04, 0.20]). Testing the four-item SPV composite, the overall effect of ambivalence did not reach significance {β = 0.09, SE = 0.05, *t*_372_ = 1.70, *P* = 0.089, 95% CI: [−0.01, 0.19]}, but it was in the same direction as our previous studies. There was no interaction with ideological polarization [β = −0.03, SE = 0.04, *t*_370_ = −0.75, *P* = 0.452] as the effect of ambivalence was in the predicted direction even when polarization was low, and the indirect effect through discomfort was significant (β = 0.11, SE = 0.03, 95% CI: [0.05, 0.18]). The proposed discomfort mechanism was therefore supported as at least an indirect contributor to SPV. With these results providing initial evidence, we examined discomfort more closely in our last two studies.

### Study 5—Real support for extreme political groups

We preregistered (https://aspredicted.org/p4h98.pdf) and conducted study 5 (*N* = 250) to assess whether ambivalence would positively predict behavioral support for real extreme political action—specifically, donations to actual pro-environmental organizations known for using extreme tactics—at least among participants with polarized pro-environmental positions because extreme attitudes likely have greater motivational potency (*55**–**56*). In contrast, we did not expect this to emerge for donations to mainstream organizations (regardless of polarization). Instead, we thought subjective ambivalence would show either a negative or null relationship with mainstream donations (as occurred with self-reported willingness in study 2). We specifically preregistered the predictions that subjective ambivalence would positively predict donations to extreme organizations at least among those with polarized positions but not to mainstream organizations. We thought that polarization could be especially important in this study because people might be more reluctant to make real donations to extreme organizations compared to self-reporting a willingness to commit extreme acts, but ambivalence could increase real extreme behavior especially among those with polarized attitudes. Thus, we again tested the effect of ambivalence both on its own and conditional on attitude polarization.

**Fig. 4.**
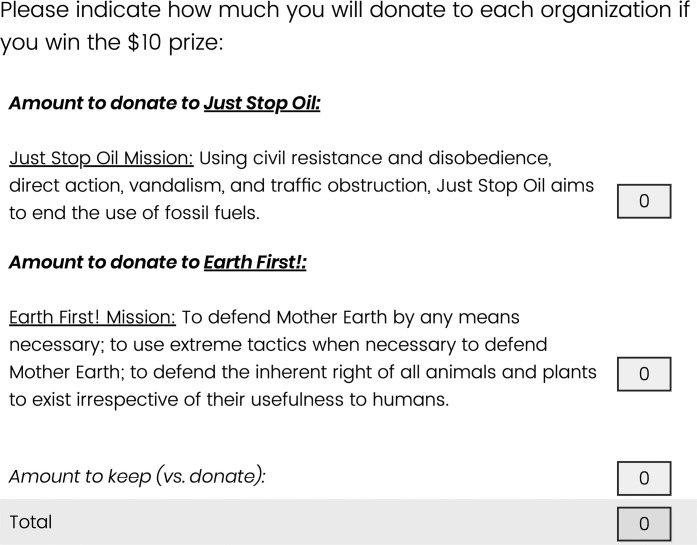
Donation to extreme environmental organizations task. The sum of the three categories was required to equal $10.

We first assessed attitudes toward and subjective ambivalence about environmentalism and then informed participants of a raffle where five random participants would receive a $10 bonus after the study. Participants were told that the purpose of the raffle was to enable them to make a real donation to an environmental charity without having to pay anything out-of-pocket. We then had participants allocate the $10 that they would have a chance to win between themselves and two real pro-environmental organizations. We told participants that the two organizations were chosen randomly from a list of 20 compiled by researchers, but in fact all participants saw two organizations well known for using extreme tactics like vandalism and road blockades (JustStopOil and EarthFirst!). These organizations’ tactics were conveyed to participants as shown in Fig. 4. Our primary dependent variable was a continuous measure of the combined amount donated to JustStopOil and/or EarthFirst! (versus the amount kept for oneself).

We also administered this task with two mainstream environmental charities (The Sierra Club and The Nature Conservancy) to assess the specificity of the effect to extreme (versus more ordinary) behavior, asking participants to allocate their potential winnings a second time to provide a backup in case we were unable to contact their initially chosen donation recipients. Last, participants reported their attitudinal discomfort [how uneasy, uncomfortable, and bothered they were with their attitude position, (*62*)] and then their willingness to commit two extreme pro-environmental acts: vandalizing the property of companies whose actions harm the environment and committing violence to protect the environment. Our goals in including these extreme behavioral willingness measures were to replicate the effect of ambivalence seen in previous studies and to validate the donation measures.

#### Validating the donation measure

We first tested the construct validity of the donation measure. Supporting the idea that participants viewed the extreme (but not mainstream) organizations as extreme, self-reported willingness to engage in extreme pro-environmental behaviors correlated with donations to extreme organizations [*r*_vandalism_(248) = 0.28, *r*_violence_(248) = 0.34, *P*s < 0.001] but not with donations to mainstream organizations [*r*_vandalism_(248) = 0.07, *P* = 0.270, *r*_violence_(248) = 0.10, *P* = 0.130]. Moreover, the extreme willingness measures also correlated with ambivalence [*r*_vandalism_(498) = 0.22, *r*_violence_(498) = 0.29, *P*s < 0.001], once again replicating the basic effect (we did not measure moderate behavioral willingness). Altogether, these relationships demonstrate the convergent and divergent validity of the extreme and moderate donation measures relative to self-reported behavioral willingness.

#### Main analyses: Ambivalence predicting donations to environmental organizations

We next tested our main hypotheses. Although on a bivariate level, ambivalence did not significantly predict donations to extreme organizations [*r*(248) = 0.03, *P* = 0.610], the relationship was significantly moderated by attitude polarization, β = 0.57, SE = 0.26, *t*_246_ = 2.19, *P* = 0.030, 95% CI: [0.06, 1.08], consistent with the effect being enhanced when the attitude is especially motivating for the person. Underlying this two-way interaction, ambivalence had a significant positive effect when attitudes toward environmentalism were highly favorable {+1 SD; β = 1.17, SE = 0.39, *t*_246_ = 2.97, *P* = 0.003, 95% CI: [0.39, 1.94]}, but not when attitudes were only moderately favorable {−1 SD; β = 0.19, SE = 0.30, *t*_246_ = 0.62, *P* = 0.538, 95% CI: [−0.41, 0.78]}. A Johnson-Neyman analysis showed that the positive effect of ambivalence was significant for the 75% of participants with attitudes at least as favorable as 7.11 (out of 9). To probe the effect further, we conducted another analysis where we standardized the extreme donation measure and the two self-reported extreme willingness measures and combined the three into an extreme behavior composite (α = 0.74). We found that ambivalence significantly positively predicted this composite [*r*(248) = 0.22, *P* < 0.001] and the ambivalence × polarization interaction was also significant {β = 0.31, SE = 0.05, *t*_246_ = 3.27, *P* = 0.001, 95% CI: [0.07, 0.27]}.

In contrast, ambivalence did not predict donations to mainstream organizations overall [*r*(248) = 0.01, *P* = 0.886] or as a function of attitude polarization {β = 0.27, *t*_246_ = 1.01, *P* = 0.312, 95% CI: [−0.26, 0.81]}, although the three-way ambivalence × polarization × donation extremity interaction, where donation extremity was treated as a within-subjects factor, did not reach significance, β = 0.29, *t*_246_ = 1.15, *P* = 0.251, 95% CI: [−0.21, 0.80]. Still, the pattern of results for extreme versus mainstream donations was consistent with our expectations: The effect of ambivalence emerged when the donation recipients were extreme, at least when attitude polarization was high, but it never emerged when the donation recipients were mainstream.

#### Mechanism: The role of discomfort

We next examined discomfort. We propose that ambivalence increases extreme behavior because such behaviors become more appealing when discomfort with one’s position is greater, whereas moderate behaviors lack that property. Thus, discomfort should differentially mediate the effects of ambivalence on donations to extreme versus mainstream environmental organizations. We first inspected simple correlations and found that in addition to a positive association with ambivalence [*r*(248) = 0.54, *P* < 0.001], discomfort was positively related to extreme donations [*r*(248) = 0.27, *P* < 0.001] but not mainstream donations [*r*(248) = 0.10, *P* = 0.122], and the discomfort-donation associations significantly differed as a function of donation extremity [*b* = 0.50, *t*(248) = 2.75, *P* = 0.006]. Discomfort was also positively associated with self-reported willingness to vandalize, *r*(248) = 0.55, *P* < 0.001, and commit violence, *r*(248) = 0.60, *P* < 0.001, in support of one’s environmental position. Next, we tested the interaction between ambivalence and attitude polarization on discomfort, which revealed a significant interaction that paralleled the effect on extreme donations, β = 0.19, SE = 0.07, *t*_246_ = 2.68, *P* = 0.008, 95% CI: [0.05, 0.33]. Greater ambivalence was associated with more discomfort even when attitudes were relatively nonpolarized {−1 SD, β = 0.63, SE = 0.08, *t*_246_ = 7.59, *P* < 0.0001, 95% CI: [0.47, 0.80]}, but the effect was significantly stronger when attitudes were highly polarized {+1 SD, β = 0.96, SE = 0.11, *t*_246_ = 8.87, *P* < 0.0001, 95% CI: [0.75, 1.18]}.

Given that the ambivalence × polarization interaction emerged for both discomfort and extreme donations, we conducted a moderated mediation analysis to test the proposed role of discomfort in mediating the effect of ambivalence at high versus low polarization. The indirect effect was significant both when polarization was high (β = 0.90, SE = 0.25, 95% CI: [0.42, 1.38]) and when polarization was low (β = 0.59, SE = 0.17, 95% CI: [0.28, 0.95]), but it was significantly larger when polarization was high, as indicated by a significant index of moderated mediation (β = 0.18, SE = 0.09, 95% CI: [0.02, 0.35]). In contrast, there was no indirect effect of ambivalence through discomfort on mainstream donations, whether polarization was high (β = 0.25, SE = 0.22, 95% CI: [−0.15, 0.71]) or low (β = 0.16, SE = 0.14, 95% CI: [−0.10, 0.46]), and the index of moderated mediation was nonsignificant (β = 0.05, SE = 0.05, 95% CI: [−0.03, 0.17]). A comparison of the indirect effects of ambivalence through discomfort on donations revealed a significant difference as a function of recipient extremity (β_diff_ = 0.45, SE = 0.14, 95% CI: [0.17, 0.73]).

In sum, study 5 replicated the positive effect of ambivalence shown in studies 1 to 4 using a measure of actual extreme behavior, namely, monetary allocations made to real environmental organizations known for using extreme tactics. Specifically, the effect emerged when attitudes were high in polarization, replicating study 2 and supporting the notion that ambivalence is especially likely to drive extreme action when the attitude about which the person is conflicted is highly motivating. However, even at low polarization, a significant indirect effect of ambivalence on extreme donations through discomfort emerged. This might indicate that the same underlying psychological process still occurred for those with less polarized attitudes, but other factors (e.g., social norms against extremism) prevented it from translating into an extreme behavioral consequence. In contrast, ambivalence did not significantly predict donations made to mainstream environmental organizations, regardless of attitude polarization, and not even indirectly through discomfort. Thus, greater ambivalence predicted greater real support for extreme political action, especially when accounting for the roles of polarization and discomfort.

### Study 6—The role of believing ambivalence is justified (versus unjustified)

So far, we have proposed that the association between ambivalence and support for extreme pro-attitudinal actions stems from ambivalence-induced discomfort. However, as noted earlier, this interpretation is compatible with two distinct processes, which we sought to examine in study 6. Specifically, ambivalence-induced discomfort could increase extreme behavior because people are trying to reduce their ambivalence or, alternatively, because people are coping with the discomfort but not aiming to reduce it. That is, rather than using extreme behaviors to help resolve the source of their discomfort, ambivalent people might be drawn to what they believe supporting extreme behaviors would signify about them. It is important to determine which of these accounts is more likely because they suggest different contexts in which the phenomenon should emerge. Thus, we conducted two experiments, 6a and 6b, testing them. Each experiment used different messages to manipulate beliefs about how justified (versus unjustified) it is to be ambivalent about political issues, in a three-cell between-subjects design (Justified versus Unjustified versus Control). If the effect results from ambivalent people trying to reduce their ambivalence, then instilling a belief that ambivalence is justified (versus unjustified) should weaken the ambivalence-reduction motive and therefore attenuate the effect. However, if the effect involves ambivalent people overcompensating for weakness when they cannot address the source of their discomfort directly, instilling a belief that ambivalence is justified (versus unjustified) should enhance the motivation to signal strength and thus the ambivalence-extremity link. We designed study 6a/b to experimentally test these alternative explanations.

In both studies, CloudResearch Connect participants (total *N* = 567) first reported their political orientation and their subjective ambivalence about their political orientation. Note that we measured political orientation in place of an attitude measure as in some study 2 samples and in study 4, and we report analyses with ideological polarization as a moderator of the ambivalence effect below. Participants then were randomly assigned to one of three conditions. In the Control condition, participants continued immediately to the discomfort measures similar to our earlier studies. In the two other conditions, participants read an article that portrayed ambivalence as either justified or unjustified. In experiment 6a, the article argued either that ambivalence is desirable (Justified ambivalence) or undesirable (Unjustified ambivalence). We then took a more indirect approach in experiment 6b, exploiting the tendency for ambivalent positions to seem more justified when the underlying evidence is two-sided (*33*). That is, in 6b, the article stated either that political issues are nuanced and involve making trade-offs (Justified ambivalence) or that they are simple and involve relatively black-and-white decisions (Unjustified ambivalence). The articles are presented in the Supplementary Materials. We asked participants to keep the article in mind as they proceeded with the study.

Participants then reported their degree of discomfort with their ideological position, followed by 15 behavioral willingness measures based on (and usually identical to) items from study 2 that were perceived as extreme or moderate on the normative behavioral extremity ratings (relative to the extremity scale’s neutral midpoint). The 15 willingness measures are identified with asterisks in Table 1. Each behavioral measure referred to a willingness to engage in a particular action in support of one’s “political beliefs” (e.g., “To what extent would you be willing to engage in violence on behalf of your political beliefs?”). Last, near the end of experiment 6a (although not 6b), we had participants evaluate political ambivalence as a manipulation check (“In your opinion, is being ambivalent about politics more a good thing or a bad thing?”). People were moderately favorable toward ambivalence in the control condition (*M* = 4.18, SD = 1.70), indicating that political ambivalence was not regarded negatively, unlike ambivalence in some other domains [e.g., racial attitudes; (*29*)]. Additionally, supporting our methodology, favorability toward ambivalence was significantly enhanced in the Justified ambivalence condition [*M* = 5.29, SD = 1.36, *b* = 1.10, *t*_281_ = 5.04, *P* < 0.0001, *d* = 0.72] and significantly reduced in the Unjustified condition [*M* = 3.38, SD = 1.57, *b* = −0.80, *t*_281_ = −3.53, *P* < 0.001, *d* = 0.49] relative to the control.

The patterns of results were similar across experiments 6a and 6b, which are the only datasets in which we manipulated how justified the ambivalence was, so we focus on combined analyses across experiments. However, we also report analyses of moderation by experiment and, when relevant, describe the results for each experiment. We first tested a multilevel model to determine whether behavioral extremity moderated the impact of ambivalence on behavioral willingness. We used composite measures of the extreme and moderate behaviors, but all conclusions held when treating behavioral extremity as a continuous moderator using the study 2 ratings. Replicating our previous studies, the ambivalence × behavioral extremity interaction was significant, β = 0.70, SE = 0.06, *t*(565) = 12.59, *P* < 0.0001, and although further moderation by experiment indicated that there was a difference in the magnitude of the ambivalence × behavioral extremity interaction across experiments [the 6a effect was larger, β = −0.29, SE = 0.12, *t*_563_ = −2.45, *P* = 0.015], it was significant within each experiment individually (*t*s > 5.4, *P*s < 0.0001).

**Fig. 5.**
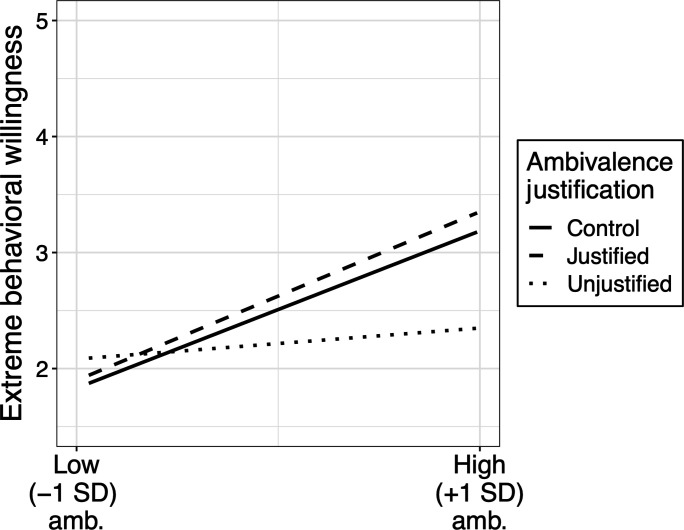
Extreme behavioral willingness as a function of ambivalence and ambivalence justification condition.

We proceeded to examine the results for extreme behaviors, shown in Fig. 5. Ambivalence was positively associated with extreme behavioral willingness overall [*r*(565) = 0.41, *P* < 0.0001], but when we regressed extreme behavioral willingness onto ambivalence and the ambivalence justification manipulation, a significant interaction emerged [*F*(2,561) = 10.67, *P* < 0.0001, η*p*^2^ = 0.019]. To better understand these results, first, we coded a new factor with two levels consisting of the Unjustified ambivalence condition (−1) and the Justified ambivalence condition (1) and regressed extreme behavioral willingness onto ambivalence and this factor. This revealed that the effect of ambivalence was significantly reduced in the Unjustified relative to the Justified condition {β = 0.30, SE = 0.07, *t*_561_ = 4.40, *P* < 0.0001, 95% CI: [0.16, 0.43]}. Additionally, the simple effect of ambivalence in the Unjustified condition was not significant overall {β = 0.13, SE = 0.09, *t*_561_ = 1.58, *P* = 0.116, 95% CI: [−0.03, 0.30]}. We then created additional new factors to compare each condition to the Control (codes are specified in parentheses). Analyses of these variables revealed that the ambivalence effect was also reduced in the Unjustified condition (−1) relative to Control (1) {β = 0.27, SE = 0.06, *t*_371_ = 4.23, *P* < 0.0001, 95% CI: [0.15, 0.40]}. In contrast, it did not differ between Justified (1) and Control (−1) {β = 0.03, SE = 0.07, *t*_561_ = 0.38, *P* = 0.706, 95% CI: [−0.11, 0.15]} and was significant and comparable in magnitude in both conditions {Justified: β = 0.73, SE = 0.10, *t*_561_ = 7.38, *P* < 0.0001, 95% CI: [0.53, 0.92]; Control: β = 0.68, SE = 0.09, *t*_561_ = 7.70, *P* < 0.0001, 95% CI: [0.51, 0.85]}.

Examining potential moderation by experiment (6a versus 6b), the interaction between ambivalence and the Unjustified (−1) versus Justified (1) factor was not moderated by experiment {β = 0.01, SE = 0.14, *t*_561_ = 0.10, *P* = 0.923, 95% CI: [−0.27, 0.30]}. However, the magnitude of the ambivalence slope in the Control condition did differ somewhat across experiments, perhaps due to random variability in baseline favorability toward ambivalence across samples. As a result, comparisons of the experimental conditions to the Control differed across studies {Justified: β = 0.28, SE = 0.13, *t*_561_ = 2.08, *P* = 0.038, 95% CI: [0.02, 0.53]; Unjustified: β = −0.26, SE = 0.13, *t*_561_ = −2.01, *P* = 0.045, 95% CI: [−0.52, −0.01]}. In 6a, the ambivalence effect was as strong in the Control condition as in the Justified condition, but in 6b, the effect in the Control condition was less strong than that in the Justified condition. Despite these fluctuations, the effect was always weakest in the Unjustified condition and strongest (or tied for strongest) in the Justified condition.

We also tested moderation of the ambivalence effect by ideological polarization, replicating the significant interaction that emerged in study 2 [β = 0.32, SE = 0.05, *t*_563_ = 7.03, *P* < 0.0001]. As before, the effect of ambivalence was significant even among ideological centrists/moderates {β = 0.28, SE = 0.07, *t*_563_ = 3.88, *P* = 0.0001, 95% CI: [0.14, 0.42]}, but it was stronger among ideological extremists {β = 0.91, SE = 0.06, *t*_563_ = 15.26, *P* < 0.0001, 95% CI: [0.79, 1.03]}. However, ideological polarization did not interact with any effects of the justification manipulation or qualify any of the interactions between ambivalence and the justification manipulation.

**Fig. 6.**
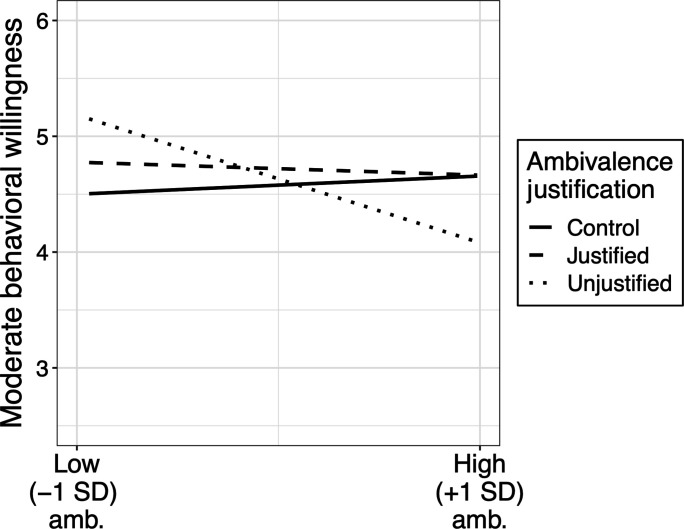
Moderate behavioral willingness as a function of ambivalence and ambivalence justification condition.

Using the same approach, we then examined moderate behaviors, shown in Fig. 6. Replicating our other studies and previous research, ambivalence negatively predicted moderate behaviors overall [*r*(565) = −0.09, *P* = 0.029], but this also depended on condition [*F*(2,561) = 10.11, *P* < 0.0001, η*p*^2^ = 0.018]. The interaction resulted from the fact that the ambivalence effect was negative and significant in the Unjustified condition {β = −0.55, SE = 0.12, *t*_561_ = −4.79, *P* < 0.0001, 95% CI: [−0.78, −0.33]} but not in the Justified condition {β = −0.06, SE = 0.09, *t*_561_ = −0.60, *P* = 0.549, 95% CI: [−0.24, 0.13]} or Control {β = 0.08, SE = 0.09, *t*_561_ = 0.89, *P* = 0.372, 95% CI: [−0.09, 0.25]}. The effect in the Unjustified condition (−1) was significantly more negative than in the Justified condition (1) {β = 0.25, SE = 0.07, *t*_561_ = 3.36, *P* < 0.001, 95% CI: [0.10, 0.39]} and the Control condition (1) {β = 0.32, SE = 0.07, *t*_561_ = 4.37, *P* < 0.0001, 95% CI: [0.17, 0.46]}. In contrast, the nonsignificant effects in the Justified (1) and Control (−1) conditions did not differ from each other {β = −0.07, SE = 0.06, *t*_561_ = −1.05, *P* = 0.295, 95% CI: [−0.19, 0.06]}. There were no significant differences in any of these patterns across experiments.

Additionally, tests of moderation by ideological polarization replicated the study 2 patterns, with the ambivalence × ideological polarization interaction significant [β = 0.09, SE = 0.04, *t*(563) = 2.35, *P* < 0.0001] and a stronger negative ambivalence effect among ideological moderates {β = −0.18, SE = 0.08, *t*_563_ = −2.36, *P* = 0.019, 95% CI: [−0.34, −0.03]} than extremists {β = 0.05, SE = 0.07, *t*_563_ = 0.74, *P* = 0.459, 95% CI: [−0.08, 0.18]}. Ideological polarization did not interact with any effects of the manipulation, however.

Finally, we examined the mediating role of discomfort. In particular, we wanted to understand whether inducing participants to consider ambivalence unjustified had reduced its impact on extreme behavioral willingness by (i) making ambivalence less uncomfortable or (ii) reducing the impact of the discomfort on willingness. In other words, which part of the process did the justification manipulation moderate—the *a* path (ambivalence → discomfort) or the *b* path (discomfort → extreme behavioral willingness)? We addressed this using moderated mediation analysis. Since both possibilities could in theory occur simultaneously, we used PROCESS model 59 (*63*) to test a model with ambivalence as the predictor, discomfort as the mediator, and Justification condition (Justified = 1, Unjustified = −1) allowed to moderate the *a* path (ambivalence → discomfort), *b* path (discomfort → extreme behavioral willingness), and *c* path (ambivalence → extreme behavioral willingness). The index of moderated mediation was significant (β = 0.54, SE = 0.16, 95% CI: [0.22, 0.85]), and the indirect effect was significant in the Justified condition (β = 0.66, SE = 0.14, 95% CI: [0.39, 0.94]) but not in the Unjustified condition (β = 0.12, SE = 0.08, 95% CI: [−0.03, 0.28]).

To fully unpack the model, we break each condition down by path. Examining the *a* path first, ambivalence predicted discomfort in both the Justified {β = 89, SE = 0.05, *t*_356_ = 19.98, *P* < 0.0001, 95% CI: [0.81, 0.98]} and Unjustified conditions {β = 0.54, SE = 0.05, *t*_356_ = 10.27, *P* < 0.0001, 95% CI: [0.44, 0.65]}. This suggests that the difference in indirect effects across conditions was not primarily due to effects on the *a* path. Instead, the overall moderated mediation pattern was largely driven by differences in effects on the *b* path. Specifically, whereas discomfort significantly predicted extreme behavioral willingness in the Justified condition {β = 0.74, SE = 0.13, *t*_356_ = 5.76, *P* < 0.0001, 95% CI: [0.49, 0.99]}, this did not occur in the Unjustified condition {*b* = 0.23, SE = 0.15, *t*_356_ = 1.47, *P* = 0.144, 95% CI: [−0.08, 0.53]}. These mediational patterns fully replicated within each experiment when analyzed separately.

In sum, making ambivalence seem unjustified (versus justified) reduced the effect of ambivalence on extreme behavioral willingness. Ambivalence was uncomfortable regardless, but the justification manipulation moderated the discomfort to willingness effect such that ambivalent people no longer translated their discomfort into support for extreme behavior when led to believe their ambivalence was unjustified. This clarifies the process underlying the phenomenon and the conditions under which it emerges. The effect does not seem to result from a motivation to reduce ambivalence. It appears instead to reflect a more self-signaling form of compensation for the discomfort ambivalent people experience.

## DISCUSSION

Evidence from 13,055 participants rating dozens of political actions varying in extremity showed that people paradoxically express greater willingness to engage in extreme forms of action and are more supportive of partisan violence (SPV) that coheres with political positions about which they feel more—not less—conflicted. This is often particularly true of more polarized attitudinal or ideological positions, indicating that felt conflict about a strongly held position is especially conducive to extreme behavior, but it emerged for self-report measures even when attitudes and ideologies were relatively low in polarization (although not actual behavior in study 5). We also replicated the well-established finding that greater ambivalence is associated with less willingness to engage in ordinary political behaviors like voting, but this simultaneously reversed for the very same ambivalence measure’s associations with people’s willingness to engage in extreme behaviors.

This finding held across methodological variations including several different topics and dozens of specific behaviors, including some that were almost maximally extreme (e.g., violence) and others that were considered somewhat extreme but less so (e.g., trying to get someone fired from their job for their political views) and that elicited a moderate level of support, on average. It also held in both archival survey data and new data we collected ourselves. The effect proved robust to confounds and alternative explanations, emerging when controlling for participants’ response tendencies—specifically, midpoint bias (the tendency to provide non-extreme responses) and acquiescence bias (the tendency to respond with agreement)—and a number of other relevant psychological and demographic variables. It was small to medium in magnitude after making these corrections (see Fig. 4). We also found that the effect was domain-specific such that political ambivalence was associated with extreme behaviors that support a person’s political views, but not unrelated extreme behaviors. Had it resulted only from response bias, then ambivalence should have positively correlated with unrelated extreme behaviors as well [see (*64*, *65*)]. Finally, the effect also emerged when measuring actual monetary donation behavior, showing that it is not merely hypothetical, but can increase support for extreme political groups in reality.

Two findings clarify the underlying process. First, we found that attitudinal discomfort plausibly generates the motivational impetus for the effect: People consistently felt greater discomfort with more ambivalent views, and this mediated their increased support for extreme actions. Attitudinal ambivalence is well known to produce discomfort, which can encourage various coping responses [e.g., (*25*–*27*)]. We show that extreme behavior is one such response. In this respect, the phenomenon parallels effects of fragile (versus secure) self-esteem, where high self-esteem that is unstable or conflicted (ambivalent) creates defensiveness and a desire to prove one’s worth (*66*). Here, the attitude being defended is not literally toward the self (i.e., self-esteem) but rather a topic with importance to the self, such as one’s political ideology. Attitudinal ambivalence regarding important or self-relevant topics, then, may suggest not just a weak attitude but a fragile attitude in need of defending with a signal of strength.

Second, we found that people’s meta-cognitive beliefs about ambivalence also matter. On the one hand, ambivalence increased extreme behavioral willingness when people believed ambivalence was justified and were therefore presumably not motivated to reduce it. On the other hand, when people were led to believe that being ambivalent was unjustified, which would presumably motivate them to reduce it, ambivalence no longer increased extreme behavioral willingness. These results indicate that an ambivalence-reduction motive is unlikely to be involved in producing the effect. Instead, it seems more like a form of “symbolic self-completion” (*34*–*35*) where ambivalent people are attracted to extreme behaviors because they help to signal or validate a desired but unattained self-image (e.g., clarity, decisiveness, and commitment). Also importantly, participants viewed ambivalence as relatively justified at baseline, and the effect of ambivalence in the justified and control conditions did not differ. These findings suggest that part of the reason the phenomenon regularly emerges is because perceiving ambivalence as justified is the norm in US politics.

To gain a more holistic understanding of the underlying psychology, it is worth considering the interplay between affective and cognitive influences in enabling ambivalence to increase people’s willingness to support or engage in extreme actions. Together, the roles of discomfort (affect) and justification (cognition) in producing this effect suggest that to increase the likelihood of the effect emerging, there should be (i) a motivational impetus for extreme action and (ii) a willingness to translate that impetus into action. Attitude polarization often enhances the motivational impetus by magnifying the discomfort from ambivalence, but the impetus can and often does exist sufficiently from ambivalence even without polarized attitudes. Therefore, a high degree of attitude polarization is not necessary for the phenomenon to emerge. Justification enhances the translation of motivation into action and does seem to be required to produce the effect, but this condition appears to be met by default in the political domain. So, although the phenomenon emerges from a particular confluence of factors (discomfort and justification), the default in the political domain is for those factors to align and therefore produce the effect.

Our findings contribute to important literatures on attitude strength [e.g., (*11*, *19*, *38*–*40*)] and political partisanship [e.g., (*1*, *41*–*44*, *67*)]. From the attitude strength standpoint, the results suggest that despite the reputation of ambivalence for promoting indecision and inaction (*7*–*9*, *14*–*18*), it can also increase the tendency to act on one’s attitudinal position, especially in extreme ways, because of the discomfort it induces. Thus, ambivalence can affect behavior through both the traditional negative route, which we replicated with moderate behaviors, and a positive route, which appears to be defensive or compensatory in nature, and which we demonstrated with extreme behaviors. In establishing this reversal, the current work joins a small but growing literature documenting the ability of properties of attitudes typically associated with strength [like certainty, (*40*, *68*)] to produce opposite effects on judgment and behavior by operating through these dual routes under different conditions.

Further, our results uncover some conditions under which negative versus positive effects of ambivalence may be more likely. As explained, negative effects emerge when people consider moderate behaviors, but positive effects become more likely for more extreme behaviors. Negative effects also emerge when ambivalence is interpreted negatively, but positive effects become more likely when ambivalence is interpreted positively. Both negative and positive effects can be magnified by other variables that indicate that the attitude is strongly held. We showed this with attitude polarization, but other attitude strength indicators like attitude certainty (*53*) could plausibly produce similar results. To test this, we conducted further analyses of our study 2 data, which also contained certainty measures. Sure enough, a multilevel model with ambivalence, certainty, and their interactions predicting extreme behavioral willingness showed the same pattern as for attitude polarization—that is, the effect of ambivalence on extreme behavioral willingness strengthened as attitude certainty increased {interaction: β = 0.17, SE = 0.02, *t*_4973_ = 8.42, *P* < 0.0001; ambivalence at low certainty: β = 0.38, SE = 0.03, *t*_4973_ = 11.82, *P* < 0.0001, 95% CI: [0.32, 0.44]; ambivalence at high certainty: β = 0.72, SE = 0.03, *t*_4973_ = 23.60, *P* < 0.0001, 95% CI: [0.66, 0.78]}. This result also aligns conceptually with study 6 in that the effect is stronger when ambivalent people consider their positions justified or valid, which certainty implies.

From the perspective of the political partisanship literature, the current findings add nuance to existing perspectives on partisan ambivalence (*1*, *7*–*9*, *41*) and could have important implications for understanding and ultimately mitigating support for partisan violence (*42*–*44*, *67*). In particular, our findings point to counterintuitive strategies for potentially reducing SPV. It seems natural to assume that encouraging people to accept that the political sphere is complex and involves trade-offs, and therefore some ambivalence about political topics is justified, might make ambivalence less uncomfortable [e.g., (*27*)], which could reduce extremism. However, this pro-ambivalence messaging strategy proved ineffective in our experiments. Instead, the opposite approach—challenging ambivalence and encouraging people to take one-sided positions—reduced extreme behavioral willingness. This anti-ambivalence message might have worked by leading ambivalent people to doubt their political positions [see (*69*–*70*)], undermining a sense that ambivalent people might hold that they have a relatively informed and unbiased perspective on the issue (*32*–*33*) and would therefore be relatively justified in taking extreme action. Notably, making ambivalence seem unjustified did not make it more comfortable, but it prevented acting upon this discomfort. Further examining the effects of these messaging approaches offers a clear opportunity to extend the present findings.

The current work is not without limitations. In particular, most of our studies are correlational, limiting our ability to draw causal conclusions. However, several features of our research make alternative causal explanations for the results less plausible than our proposed process whereby attitudinal ambivalence increases extreme pro-attitudinal behavior. We found that manipulating the perceived justification for ambivalence after measuring ambivalence changed its effect on willingness to engage in extreme behavior, which a reverse causal model (i.e., extremism increasing ambivalence) struggles to explain. As for third variable explanations (i.e., some other variable increasing both ambivalence and extremism), these, too, were ultimately not well supported in our data. For one thing, if some variable other than ambivalence is responsible for increased extreme behavior, then it is unclear why messaging about ambivalence would moderate the effect. The fact that the effect emerged using both subjective and objective measures of ambivalence, which are operationalized completely differently, is also hard to square with a third variable explanation. Also notably, the effect was robust to controlling for response biases and various other relevant psychological and demographic covariates, making it unlikely that these variables are responsible. We believe that the data are therefore more consistent with the causal model we have proposed, namely, that attitudinal ambivalence, when considered justified, increases support for extreme political action.

Another question is the generalizability of the effect of ambivalence on extremism beyond the political domain. Ambivalence about any topic that matters to a person can be uncomfortable (*25*–*26*), so perhaps similar effects to what we observed for political behavior could emerge elsewhere. For example, feeling conflicted about one’s romantic relationship might promote extreme forms of pro-relationship behavior, at least when one is committed to the relationship. However, it is unclear whether this generally occurs. Our findings regarding the perceived justification for ambivalence suggest that the effect is less likely when people do not want to be ambivalent, as is likely often true in a relationship context. Politics might be relatively unusual in that many people believe that it is legitimately two-sided (or multi-sided) and therefore that ambivalence and compromise about political issues are justified and appropriate, though still uncomfortable.

Relatedly, it is also interesting to consider potential cross-cultural differences in these processes. For example, would the effect of ambivalence on extreme behavior be more prevalent among populations who more often believe ambivalence is justified, such as those high in dialectical thinking, or would it be less prevalent in this population due to their lower discomfort with ambivalence (*71*)? Ultimately, the effect could plausibly emerge whenever people feel uncomfortably yet justifiably ambivalent about political or nonpolitical issues.

Finally, we do not view our findings as an indictment of political ambivalence or compromise. As noted, previous work on ambivalence also finds associations with characteristics many would regard as desirable including a deliberative mindset about political issues (*1*) and less susceptibility to cognitive bias (*6*). Of course, ambivalence also tends to reduce voting and other forms of democratic participation (*7*–*9*). Our findings about ambivalence and extreme behavior seem compatible with these other tendencies, but it remains to be seen whether and to what extent they co-occur in the same people, and if so, how they fit together psychologically. Notably, the effect was enhanced when people’s attitudinal or ideological positions were more polarized, so the phenomenon clearly does not indicate that political centrists/moderates are actually covert extremists. Also, we examined extreme behaviors that are generally socially disapproved, but the same processes could plausibly extend to more acceptable extreme behaviors, including some that are considered highly desirable and even morally exceptional or heroic (*12*). For example, political ambivalence might also increase willingness to engage in extreme prodemocratic behaviors (e.g., devoting all one’s time and resources to solving an important societal problem) as these also provide signal value. The behaviors we examined were intended to vary in extremity but not to represent all relevant behaviors, so it would be valuable to test effects of ambivalence on actions that are prosocial, prodemocratic, etc.

As political polarization and conflict intensify, political extremism is more salient in America and around the world than it has been in decades. We believe that taking a belief-based perspective that accounts for the roles of ambivalence, ambivalence-induced discomfort, and the perceived justification for being ambivalent in driving support for extreme political action contributes to an emerging understanding of how psychological factors can make political extremism attractive. We also hope that targeting these processes might offer generative strategies for developing anti-extremism interventions going forward.

## MATERIALS AND METHODS

These studies were approved by the Office of Responsible Research Practices at The Ohio State University.

### Study 1

VOTER is a national survey of Americans administered by the Democracy Fund Voter Study Group and YouGov. Between 22 November and 2 December 2019, VOTER sampled 5900 respondents (*M*_age_ = 52.02, SD_age_ = 16.81; female = 3076, male = 2824). We calculated our partisan ambivalence measure using the well-established similarity-intensity method (*4*–*5*, *7*–*8*, *41*) with the feeling thermometer ratings of the Democratic (*M* = 49.82, SD = 35.47) and Republican (*M* = 44.56, SD = 34.25) parties (i.e., the measures’ average minus the absolute value of their difference). Higher values on this measure reflect greater simultaneous positivity toward both parties. Four SPV measures were taken—how justified respondents thought it was for members of their political party to use violence to advance their political goals (five-point scale, 1 = not at all, 2 = a little, 3 = a moderate amount, 4 = a lot, 5 = a great deal; *M* = 1.26, SD = 0.79) and specifically in the event of the opposing party winning the then-upcoming 2020 presidential election (*M* = 1.39, SD = 0.98), how often it was justified to threaten politicians belonging to the opposing party (four-point scale, 1 = never, 2 = occasionally, 3 = frequently, 4 = always; *M* = 1.31, SD = 0.74) and harass members of the opposing party on the internet (*M* = 1.26, SD = 0.71). Respondents also reported whether they intended to vote in the then-upcoming 2020 election (rescaled so 1 = no, 2 = not sure, 3 = yes; *M* = 2.86, SD = 0.44). Our analyses used the weighting variable provided by VOTER, but the results are substantively unchanged when not using the survey weight. Finally, perceived out-party intentions were assessed with this prompt: “Regardless of what you think about the policies they favor, do you think the following groups have mostly good or mostly bad intentions?” The question was posed twice: once referring to “Leaders of the Democratic/Republican party” and once to “People who vote for Democratic/Republican candidates” (1 = mostly good intentions, 5 = mostly bad intentions).

CES is a national survey administered by YouGov during election years in the US. In addition to “Common Content” asked of all respondents, CES fields a number of *n* = 1000 “Team Content” surveys among subsamples that cover specific content of interest to the organizing team. A team of Louisiana State University and University of Maryland researchers fielded a survey between September and December 2020 that served as the basis of our analyses (*M* = 48.17, SD_age_ = 17.75; female = 532, male = 468) (*43*). As in VOTER, partisan ambivalence was calculated using the feeling thermometer ratings of the Democratic (*M* = 48.57, SD = 35.18) and Republican (*M* = 38.62, SD = 34.62) parties. Intentions to vote in the then-upcoming 2020 presidential election and self-reported political participation over the past year were also included in Common Content. The same four SPV items were measured as in VOTER. To construct a linear response scale for voting intentions, we recoded responses as follows: “yes, definitely” = 4, “I already voted (early or absentee)” = 4, “I plan to vote before November 3^rd^” = 4, “probably” = 3, “undecided” = 2, “no” = 1 (*M* = 3.48, SD = 1.01). Trait aggression was assessed with four items: “Given enough provocation, I may hit a person,” “My friends say I am somewhat argumentative,” “I have trouble controlling my temper,” and “At times, I feel I have gotten a raw deal out of life.” (1 = Completely false for me, 7 = Completely true for me; α = 0.65). A political knowledge measure was constructed from 0 to 4 based on the number of correct answers provided to queries about the party that controlled the Presidency, Senate, House, and Supreme Court based on the party that appointed the Justices; *M* = 3.42, SD = 0.85). Partisan social identification was assessed with three items: “How important is being (your party insert) to you?,” “When talking about (your party insert) how much do you use “we” instead of “they”?,” and “To what extent does being (your party insert) make you feel important?” (reverse-scored for analysis such that 1 = Not at all, 4 = Very much, α = 0.76).

We also checked other publicly available surveys for suitable measures, namely, measures of support for extreme forms of pro-attitudinal political action. Notably, although the 2020 American National Election Studies survey included an item measuring supporting political violence (V201602), it was not specified to be pro-attitudinal. Our framework predicts effects on extreme behaviors that support one’s position, but not extreme behaviors that are unrelated or oppose one’s position, which are also captured by the American National Election Studies measure.

#### Response biases

We estimated and controlled for response biases in our analyses. It seemed especially important to examine the tendency to provide responses near the scale midpoint (“midpoint bias”) since ambivalence has been tied to reporting moderate opinions in previous research (*46*). More generally, controlling for response biases helps address the potential for inattentive responding to artifactually inflate statistical relationships (*64*–*65*). With that concern in mind, in addition to midpoint bias, we also examined the tendency to agree regardless of question content (acquiescence bias). In VOTER, we computed these two response bias measures using 19 items where participants rated the degree to which different ideological and group labels applied to them (e.g., environmentalist, libertarian, moderate, alt-right, and feminist). In CES, we computed the response bias measures using 12 items where participants rated their perception of the political ideology of different targets (e.g., Biden, Trump, and the respondent’s state’s governor and senators). In each dataset, the measures we used to estimate response biases should elicit widely varying responses from each participant, so averaging across them can provide an estimate of how people respond regardless of question content. We calculated the measures’ extremity for midpoint bias (i.e., the absolute value of the difference between the response and the scale midpoint) and their mean for acquiescence bias. To the extent that a person’s responses are similar or similarly extreme across questions, this would indicate that they are likely not paying close attention or are otherwise disengaged from the content they are being asked about. Addressing this possibility, the results controlling for midpoint bias are shown in the manuscript, and our conclusions also held when controlling for acquiescence bias instead of or in addition to midpoint bias. Specifically, in CES, ambivalence still had a significant effect on the same three SPV measures, and in VOTER, two of the four effects remained significant with both response biases controlled (agreeing that partisan violence and threatening out-party members online are justified).

### Study 2

We compiled 19 datasets we had previously collected over about 3 years (*N* = 5082, *M*_age_ = 35.76, SD_age_ = 14.02; female = 2583, male = 2467, other/prefer not to say/missing = 32). Of these, 16 were collected on Mechanical Turk using the CloudResearch platform (*72*), using all available precautions to ensure data quality that were available on CloudResearch at the time of each data collection. The other three datasets were collected from undergraduate students participating for course credit. [A subset of these datasets was also used for a different, nonoverlapping set of analyses reported in (*40*).] Participants in these and all our studies provided their informed consent before enrolling. Because the behavioral intention measures varied across studies, the sample size varied across analyses of the behaviors (see Table 1). Sensitivity analyses (α = 0.05, two-tailed) showed that our main analyses provided 0.80 (0.99) statistical power to detect correlation effect sizes of *r* = |0.26| (*r* = |0.39|) for the behaviors with the least observations (washing hands/following COVID-19 guidelines; *n* = 110) and just *r* = |0.04| (*r* = |0.06|) for the behavior with the most observations (fighting; *n* = 4909). Subjective ambivalence (*M* = 2.74, SD = 1.80) was assessed with three items measuring how mixed, conflicted, and indecisive participants felt about their attitude (*4*). Objective ambivalence about the same attitude object (seven samples, *n* = 1702; *M* = 1.73, SD = 2.74) was assessed with two items. The first item measured positive feelings toward the attitude object while “ignoring the negatives,” and the second measured negative feelings while “ignoring the positives” [seven-point scales; (*3*)]. We used these two measures to create an ambivalence index via the similarity-intensity formula [their average minus the absolute value of their difference, e.g., (*4*–*5*, *7*–*9*, *14*, *44*)].

Some samples also included measures we tested as covariates, namely, attitude certainty and political ideology. Attitude certainty is a widely studied attitude property that is generally associated with both ambivalence and attitudes’ impact on behavior (*38*–*39*), so it was important to determine whether the effect of ambivalence is independent of any effects of certainty. Political ideology was recently found to be related to ambivalence across a range of attitude objects (*45*, *54*), so it too seemed relevant to examine as a covariate. To test robustness, we conducted reanalyses controlling for these variables as well as response biases (described below).

#### Behavioral willingness measures

We examined 27 behavioral willingness measures across the 19 samples. Most measures used identical language when measured in multiple studies, but for several variables, we combined versions of the same measure with different wordings (shown in Table 1). This is spelled out in Table 1 with the exception (due to space) of willingness to sacrifice one’s life for one’s position. In four samples, this was measured with a five-item scale [from (*73*); see the Supplementary Materials]. In two samples, it was measured with two items: “To what extent would you be willing to sacrifice your life…” either “if it helped find a vaccine against COVID-19?” or “to COVID-19 if it helped bring the economy back sooner?” For all measures, the different wordings always independently produced directionally consistent results. All measures used seven-point scales (1 = not at all, 7 = very much). Table S1 presents all items and their correlations with subjective ambivalence broken down by sample. (Note that we used different wording for the martyrdom measure in study 6: “To what extent would you be willing to sacrifice your life on behalf of your political beliefs?”)

#### Normative behavioral extremity ratings

We recruited an additional sample (*N* = 426, *M*_age_ = 41.84, SD = 12.72, female = 214) to provide extremity ratings of the 26 behaviors (“How extreme is [X]?”; seven-point scales; 1 = not at all, 7 = very much). Each behavior received 69 to 72 ratings.

#### Behavioral directionality

Some behavioral measures imply a favorable evaluation of one position (i.e., “directional” behaviors, such as avoiding crowds, which implies a favorable evaluation of social distancing), whereas others apply generically to whatever participants’ position happens to be (i.e., “nondirectional” behaviors such as voting for a political candidate). For analyses of directional behaviors, we excluded the relatively few participants for whom the behavior was attitude-inconsistent (e.g., who opposed social distancing), although analyzing data from all participants leaves the results largely unchanged. For nondirectional behaviors, we analyzed all participants without exclusions.

#### Response biases

Although our multilevel analyses model individual participants from all studies, so the main results should account for participant-level response tendencies, we nonetheless sought to directly address the possibility that response biases could contribute to the results. We explained our reasoning for doing so in study 1. Using the same approach as in study 1 (with different measures, based on availability), we constructed midpoint and acquiescence indices by taking all of the behavioral willingness measures that were included in a given study and calculating their extremity for midpoint bias (i.e., the absolute value of the difference between the response and the scale midpoint) and their mean for acquiescence bias. We then repeated our analyses while statistically controlling for midpoint and acquiescence biases, as described.

### Study 3

We recruited 250 Prolific workers using a prescreener to limit participation to those currently in a romantic relationship and excluded 6 who failed the validation screener (i.e., who said they were not in a relationship). A sensitivity analysis (α = 0.05, two-tailed) showed that our Amazon Mechanical Turk sample of 244 (*M*_age_ = 44.83, SD_age_ = 14.39; women = 161, men = 76, nonbinary = 3, other = 4) provided 0.80 power to detect an association between subjective ambivalence (measured the same way in studies 2 to 6, *M* = 2.44, SD = 1.57) and the behavioral willingness measures of *r* = |0.18| or larger. The four willingness measures were: *To what extent would you be willing to*… (i) *devote an hour of your time to benefit your romantic relationship/causes aligned with your political beliefs and opinions?* (relationship: *M* = 6.43, SD = 1.17; political: *M* = 4.17, SD = 1.90), (ii) *spend a modest sum of money to benefit your romantic relationship/causes aligned with your political beliefs and opinions?* (relationship: *M* = 5.53, SD = 1.78; political: *M* = 3.27, SD = 1.84), (iii) *aggressively confront someone who was attacking your romantic relationship/political beliefs and opinions?* (relationship: *M* = 4.22, SD = 2.16; political: *M* = 2.11, SD = 1.54), and (iv) *engage in violence to defend your romantic relationship/political beliefs and opinions?* (relationship: *M* = 3.07, SD = 2.16; political: *M* = 1.53, SD = 1.12).

### Study 4

A sensitivity analysis (α = 0.05, two-tailed) showed that our Amazon Mechanical Turk sample of 374 (*M*_age_ = 39.40, SD_age_ = 13.04; women = 227, men = 144, other = 3) provided 0.80 power to detect associations between subjective ambivalence (*M* = 2.43, SD = 1.54) and support for partisan violence (violence today: *M* = 1.64, SD = 1.21; election violence: *M* = 1.54, SD = 1.15; threatening politicians: *M* = 1.49, SD = 1.16; online harassment: *M* = 1.43, SD = 1.04) of *r* = |0.14|.

### Study 5

A sensitivity analysis (α = 0.05, two-tailed) showed that our Amazon Mechanical Turk sample of 250 (*M*_age_ = 40.87, SD_age_ = 13.41; women = 109, men = 140, nonbinary = 1) provided 0.80 power to detect associations between subjective ambivalence (*M* = 2.49, SD = 1.58) and extreme donations (*M* = 3.07, SD = 3.89) or moderate donations (*M* = 5.06, SD = 4.05) of *r* = |0.18|.

### Study 6

We recruited 304 participants in study 6a, and to ensure successful delivery of our manipulation (and attentiveness more generally), we excluded 20 who failed a simple manipulation attention check (“Thinking back to the article you read earlier, did the article state that being ambivalent about politics is more a good thing or a bad thing?”). Similarly, we recruited 303 participants in 6b and excluded 21 who failed the attention check (“Thinking back to the article you read earlier, did the article state that political issues are more simple or more complex?”). We collected both samples via CloudResearch Connect (*72*). A sensitivity analysis (α = 0.05, two-tailed) showed that our final sample of 567 (*M*_age_ = 41.94, SD_age_ = 12.72, women = 274, men = 289, other/prefer not to say/missing = 4) provided 0.80 power to detect interactions between subjective ambivalence (*M* = 2.63, SD = 1.73) and the ambivalence justification manipulation on extreme behavioral willingness (α = 0.91; *M* = 2.49, SD = 1.40) or moderate behavioral willingness (α = 0.90; *M* = 4.67, SD = 1.35) of η*p*^2^ = 0.017. The manipulations are presented in full in the Supplementary Materials.

#### Behavioral willingness measures

We measured 15 behavioral willingness measures from study 2 (denoted with asterisks in Table 1), 7 extreme and 8 moderate. For reference, five of the extreme behavioral willingness measures read, “To what extent would you be willing to…” either “get into a heated argument with someone if they attacked your political beliefs?”; “block on all social media and discontinue all contact with someone who disagrees with your political beliefs?”; “aggressively confront someone who was acting in a way that is opposed to your political beliefs?”; “engage in violence on behalf of your political beliefs?”; or “sacrifice your life on behalf of your political beliefs?” Another two extreme behavioral willingness measure behaviors read, “If someone was behaving in a way that is opposed to your political beliefs, to what extent would you be willing to…” either “try to get them fired from their job?” or “fight them?” All eight moderate behavioral willingness measures read, “To what extent would you be willing to…” either “read newspaper articles that support your political beliefs?”; “vote for political candidates who agree with your political beliefs?”; “support political candidates who share your political beliefs?”; “put effort into supporting your political beliefs?”; “volunteer your time to support causes that agree with your political beliefs?”; “donate money to causes that support your political beliefs?”; “advocate to others on behalf of your political beliefs?”; or “publicly advertise your political beliefs (e.g., via social media, a bumper sticker, t-shirt, etc.)?”
