# Supplementary material for: Ambivalent attitudes promote support for extreme political actions
Source: Sci Adv. 2024 Jun 12;10(24):eadn2965. doi: 10.1126/sciadv.adn2965 (PMC11168463; doi:10.1126/sciadv.adn2965)
Supplement: Supplementary file 1 — Supplementary Text Table S1 [file sciadv.adn2965_sm.pdf]

Science Advances
AAAS

Supplementary Materials for

Ambivalent attitudes promote support for extreme political actions

Joseph J. Siev and Richard E. Petty

Corresponding author: Joseph J. Siev, sievj@darden.virginia.edu

Sci. Adv. **10**, eadn2965 (2024)
DOI: 10.1126/sciadv.adn2965

This PDF file includes:

Supplementary Text
Table S1

Study 6 Ambivalence is Justified vs. Unjustified Manipulations

Justified ambivalence article, Study 6a:

In the current political landscape, it is common to feel pressure to choose a side and remain steadfast in one's beliefs. However, an appreciation of the benefits of ambivalence, which refers to holding both positive and negative feelings towards a particular issue or political view, suggests that it is normal to have mixed and at times conflicting political opinions. Ambivalence can be good for both the individual who feels that way and the political process as a whole. Advocates of this perspective argue that feeling ambivalent about political issues indicates a nuanced and well-considered approach to decision-making. Ambivalent individuals are not bound by ideology or partisan politics, but instead consider the pros and cons of both sides of an argument when forming an opinion. This approach leads to fairer and more impartial decision-making, as individuals are not swayed by preconceived notions or political agendas.

Furthermore, holding ambivalent views demonstrates a motivation to understand the truth. Individuals who feel conflicted about their political views are likely to engage in critical thinking, researching and evaluating information to arrive at well-informed conclusions. This approach cultivates a more informed and engaged citizenry, as individuals are not simply accepting information without questioning its validity.

In conclusion, feeling ambivalent about one's political views should not be viewed as a weakness or a failure to take a stance. Instead, it is often a sign of a nuanced, well-considered, and truth-seeking approach to decision-making about political issues.

Unjustified ambivalence article, Study 6a:

In the current political landscape, it is important to have a clear and consistent commitment to one's beliefs. However, for individuals who feel mixed or conflicted about their political views,

this clarity is elusive. This is unfortunate because ambivalence, which refers to holding both positive and negative feelings towards a particular issue or political view, can have negative consequences for both the individual who feels that way and the political process as a whole. Critics argue that holding ambivalent views demonstrates a lack of conviction and commitment. Ambivalent individuals seem indecisive and unreliable, making it difficult for others to trust their judgment. Furthermore, ambivalence can be perceived as, and may often truly reflect, a lack of dedication. This can lead to a decline in trust and support from others. As a result, ambivalent people are limited in how much influence they can have on others.

Furthermore, feeling ambivalent about one's political views can also lead to confusion and disengagement. When individuals are unable to make a clear decision, they may become disillusioned or uninterested in the political process, making them less likely to vote and engage in other political activities. This undermines the effectiveness of politics and can lead to apathy and disengagement among the general population.

In conclusion, it is essential for individuals to have a clear and consistent commitment to their political beliefs. Ambivalence can undermine the credibility of the individual and cause the political process as a whole to stagnate due to indecision and disengagement.

Justified ambivalence article, Study 6b:

Politics is viewed by some as being reducible to relatively simple, black-and-white problems that have clear solutions. However, the truth is that many political issues are not as simple as they are made out to be. There are rarely clear-cut answers or perfect solutions to political issues, and often the choices we face are not between objectively right versus wrong. In fact, many political issues involve choices between different shades of grey.

This is because political issues are often entangled with a web of competing interests, values,

and perspectives. What may be beneficial for one group or goal may come at the expense of others, and finding a fully satisfying solution is often a challenging task because in some situations, each side has a valid point to make.

Therefore, although politics is sometimes portrayed as being black-and-white, it is essential to understand that political issues are not straightforward, and there are seldom easy answers or simple solutions. Politics is a complex landscape of competing interests, values, and perspectives that often involves negotiating between tradeoffs and imperfect solutions.

Unjustified ambivalence article, Study 6b:

Politics is viewed by some as a complex and multifaceted arena that requires careful consideration of competing interests and values. However, the truth is that many political issues are not as complicated as they are made out to be. Political issues often have relatively clear-cut, straightforward answers, and the choices we face are sometimes between objectively right versus wrong. In fact, many political issues can be reduced to relatively simple, black-and-white problems that actually have clear solutions.

This is because political issues are not always about competing interests, values, or perspectives. While some issues sometimes require compromise between different groups or objectives, often the right solution is simply obvious to anyone who looks closely at the issue. That is, in some situations, one side clearly has the more valid points to make.

Therefore, although politics is sometimes portrayed as being nuanced and complex, it is essential to understand that many political issues are not actually as complicated as they might seem. Some issues are straightforward and offer clearly good solutions that should be agreed upon by everyone.

Study 2 Measures and Results by Sample

SAMPLE	*TOPIC*	*AMBIV. MEASURE*	*N*	*EXTREM.*	*BEHAVIORS*	*BEHAVIORAL WILLINGNESS ITEMS*	*AMBIV.-BEH. CORRELATION*
1	Gun control	"…about gun control?"<br><br>Measured **before** extreme behavioral willingness	202	Extreme	Fight opponents	I would fight someone who was attacking the [pro gun control/pro gun rights] position.<br><br>I would fight someone insulting or making fun of the [X] position.<br><br>I would help others get revenge on someone who insulted the [X] position.<br><br>I would be willing to hurt other people if it helped protect the [X] position.<br><br>I'd do anything to protect the [X] position.	*r*(200) = .50, [.38, .59]
			98		Sacrifice life	I would sacrifice my life if it helped preserve the [X] position.<br><br>I would sacrifice my life if it gave the [X] position status or monetary reward.	*r*(96) = .23, [.03, .41]
2	Abortion legality	"…about legal abortion?"<br><br>Measured **before** extreme behavioral willingness	201	Extreme	Fight opponents	*Same items as Study 1, referring to "the pro-choice/pro-life position"*	*r*(199) = .68, [.60, .75]
			199		Sacrifice life	*Same items as Study 1, referring to "the pro-choice/pro-life position"*	*r*(197) = .71, [.63, .77]

3	National identity	"…about your country?"<br><br>Measured **before** extreme behavioral willingness	200	Extreme	Fight opponents	*Same items as Study 1, referring to "my country"*	$r(198) = .23$, [.09, .35]
					Sacrifice life	*Same items as Study 1, referring to "my country"*	$r(198) = .24$, [.11, .37]
4	Ethnic identity	"…about your ethnic group?"<br><br>Measured **before** extreme behavioral willingness	200	Extreme	Fight opponents	*Same items as Study 1, referring to "my ethnic group"*	$r(198) = .32$, [.19, .44]
					Sacrifice life	*Same items as Study 1, referring to "my ethnic group"*	$r(198) = .42$, [.30, .53]
5	Gun control	"…about gun control?"<br><br>Measured **before** extreme behavioral willingness	201	Extreme	Fight opponents	*Same items as Study 1*	$r(199) = .47$, [.35, .57]
			200		Sacrifice life	*Same items as Study 1*	$r(198) = .38$, [.26, .49]
6	COVID social distancing	"…about social distancing?"<br><br>Measured **before** extreme behavioral willingness	299	Extreme	Fight opponents	If someone was behaving in a way that is opposed to your views about social distancing, to what extent would you be willing to fight them?	$r(297) = .62$, [.54, .68]
					Sacrifice life for vaccine	To what extent would you be willing to sacrifice your life if it helped find a vaccine against COVID-19?	$r(297) = .69$, [.62, .74]
					Sacrifice life for economy	To what extent would you be willing to sacrifice your life to COVID-19 if it helped bring the economy back sooner?	$r(297) = .75$, [.69, .79]
					COVID vaccine trial	To what extent are you willing to	$r(297) = .65$, [.58, .71]

						participate in an experimental clinical trial of a previously untested vaccine against COVID-19?	
					COVID treatment trial	If you became sick with COVID-19, to what extent would you be willing to participate in an experimental clinical trial of a new and previously untested treatment for the illness?”	*r*(297) = .45, [.35, .54]
					Self-isolation (COVID)	To what extent are you willing to completely isolate yourself for the next year from all other people you do not live with to help prevent the spread of COVID-19?	*r*(297) = .25, [.14, .35]
				Moderate	Avoid crowds (COVID)	To what extent are you willing to avoid crowds of 50 or more people for the next month to help prevent the spread of COVID-19?	*r*(297) = -.40, [-.49, -.30]
					Get vaccinated (COVID)	To what extent are you willing to get vaccinated against COVID-19 when the vaccine becomes available?	*r*(297) = -.05, [-.16, .07]
					Get treated (COVID)	If you became sick with COVID-19, to what extent would you be willing to take the first newly developed and approved medications for the illness?	*r*(297) = .18, [.06, .28]

				N/A	Change opponents' behavior	If someone was behaving in a way that is opposed to your views about social distancing, to what extent would you be willing to try to change their mind?	*r*(297) = .17, [.06, -.28]
7	COVID mask mandate	"…about mask wearing requirements?"<br><br>Measured **before** extreme behavioral willingness	401	Extreme	Fight opponents	If someone was behaving in a way that is opposed to your views about mask wearing requirements, to what extent would you be willing to fight them?	*r*(399) = .41, [.33, .49]
					Commit violence	To what extent would you be willing to engage in violence on behalf of your position on mask wearing requirements?	*r*(399) = .50, [.42, .57]
					Sacrifice life for vaccine	To what extent would you be willing to sacrifice your life if it helped find a vaccine against COVID-19?	*r*(399) = .40, [.32, .48]
					Sacrifice life for economy	To what extent would you be willing to sacrifice your life to COVID-19 if it helped bring the economy back sooner?	*r*(399) = .53, [.45, .59]
					COVID vaccine trial	To what extent are you willing to participate in an experimental clinical trial of a previously untested vaccine against COVID-19?	*r*(399) = .23, [.14, .32]
					COVID treatment trial	If you became sick with COVID-19, to	*r*(399) = .11, [.02, .21]

						what extent would you be willing to participate in an experimental clinical trial of a new and previously untested treatment for the illness?"	
					Self-isolation (COVID)	To what extent are you willing to completely isolate yourself from all other people you do not live with until the pandemic is over to help prevent the spread of COVID-19?	*r*(399) = -.07, [-.17, .03]
					Get opponents fired	To what extent would you be willing to try to get someone who disagrees with you about mask wearing requirements fired from their job?	*r*(399) = .32, [.23, .40]
					Block opponents	To what extent would you be willing to block on all social media and discontinue all contact with someone who disagrees with you about mask wearing requirements?	*r*(399) = .06, [-.04, .15]
				Moderate	Wear mask (COVID)	To what extent are you willing to wear a mask in public to help prevent the spread of COVID-19?	*r*(399) = -.15, [-.24, -.05]
					Advocate	To what extent are you willing to try to persuade others who disagree with you about mask wearing requirements?	*r*(399) = -.14, [-.24, -.04]

					Avoid crowds (COVID)	To what extent are you willing to avoid large crowds of people to help prevent the spread of COVID-19?	*r*(399) = -.16, [-.25, -.06]
					Political support	To what extent are you willing to support political candidates who share your views on mask wearing requirements?	*r*(399) = -.19, [-.28, -.09]
					Get vaccinated (COVID)	To what extent are you willing to get vaccinated against COVID-19 when the vaccine becomes available?	*r*(399) = -.03, [-.13, .06]
					Get treated (COVID)	If you became sick with COVID-19, to what extent would you be willing to take the first newly developed and approved medications for the illness?	*r*(399) = .05, [-.05, .15]
				N/A	Change opponents' behavior	If someone was behaving in a way that is opposed to your views about mask wearing requirements, to what extent would you be willing to try to change their mind?	*r*(399) = -.08, [-.18, .01]
8	Covid mask mandate	"…about mask wearing requirements?"<br><br>Measured **before** extreme	124	Extreme	Fight opponents	If someone was behaving in a way that is opposed to your views about mask wearing requirements, to what extent would you be willing to fight them?	*r*(122) = .22, [.05, .39]

		behavioral willingness			Commit violence	To what extent would you be willing to engage in violence on behalf of your position on mask wearing requirements?	*r*(122) = .37, [.21, .52]
					Confront opponents	To what extent would you be willing to aggressively confront someone who was acting in a way that is opposed to your views about mask wearing requirements?	*r*(122) = .11, [-.07, .28]
					Argue with opponents	To what extent would you be willing to get into a heated argument with someone if they attacked your political beliefs and opinions?	*r*(122) = .05, [-.12, .23]
			126	Moderate	Wear mask (COVID)	To what extent are you willing to wear a mask in public to help prevent the spread of COVID-19?	*r*(124) = -.09, [-.26, .09]
					Avoid crowds (COVID)	To what extent would you be willing to avoid large crowds of people to help prevent the spread of COVID-19?	*r*(124) = -.17, [-.34, .00]
					Wash hands (COVID)	To what extent would you be willing to wash or sanitize your hands regularly when outside your home to help prevent the spread of COVID-19?	*r*(124) = -.28, [-.43, -.11]

					Follow COVID guidelines	To what extent would you be willing to follow recommendations regarding health precautions during the COVID-19 pandemic?	$r(124) = -.18$, [-.34, -.00]
9	Political orientation	"…about your political approach?"<br><br>Measured **after** extreme behavioral willingness	300	Extreme	Fight opponents	If someone was behaving in a way that is opposed to your political approach, to what extent would you be willing to fight them?	$r(298) = .31$, [.20, .41]
					Commit violence	To what extent would you be willing to engage in violence on behalf of your political approach?	$r(298) = .32$, [.22, .42]
					Confront opponents	To what extent would you be willing to aggressively confront someone who was acting in a way that is opposed to your political approach?	$r(298) = .08$, [-.03, .19]
					Argue with opponents	To what extent would you be willing to get into a heated argument with someone if they attacked your political beliefs and opinions?	$r(298) = .05$, [-.07, .16]
				Moderate	Vote	To what extent would you be willing to vote for political candidates who agree with your political approach?	$r(298) = -.32$, [-.42, -.21]
					Advocate	To what extent would you be	$r(298) = -.32$, [-.42, -.21]

						willing to advocate for your political approach?	
					Donate money	To what extent would you be willing to donate money to causes that support your political approach?	*r*(298) = -.14, [-.25, -.03]
					Volunteer	To what extent would you be willing to volunteer your time to support causes that agree with your political approach?	*r*(298) = -.16, [-.27, -.05]
10	Political orientation	"…about your political beliefs and opinions?" <br>Measured **before** extreme behavioral willingness	303	Extreme	Fight opponents	If someone was behaving in a way that is opposed to your political beliefs and opinions, to what extent would you be willing to fight them?	*r*(301) = .36, [.26, .45]
					Commit violence	To what extent would you be willing to engage in violence on behalf of your political beliefs and opinions?	*r*(301) = .39, [.29, .48]
					Confront opponents	To what extent would you be willing to aggressively confront someone who was acting in a way that is opposed to your political beliefs and opinions?	*r*(301) = .27, [.16, ,37]
					Argue with opponents	To what extent would you be willing to get into a heated argument with someone if they attacked your	*r*(301) = .11, [-.00, .22]

						political beliefs and opinions?	
					Get opponents fired	If someone was behaving in a way that is opposed to your political beliefs and opinions, to what extent would you be willing to try to get them fired from their job?	*r*(301) = .35, [.25, .44]
				Moderate	Vote	To what extent would you be willing to vote for political candidates who share your political beliefs and opinions?	*r*(301) = -.25, [-.36, -.15]
					Advocate	To what extent would you be willing to advocate to others on behalf of your political beliefs and opinions?	*r*(301) = -.12, [-.23, -.00]
					Donate money	To what extent would you be willing to donate money to causes that support your political beliefs and opinions?	*r*(301) = -.02, [-.13, .09]
					Display views	To what extent would you be willing to publicly advertise your political beliefs and opinions (e.g., via social media, a bumper sticker, t-shirt, etc.)?	*r*(301) = -.06, [-.17, .05]
11	Mask mandate	"…about mask wearing requirements?"	209	Extreme	Fight opponents	If someone was behaving in a way that is opposed to your views about mask wearing requirements, to	*r*(207) = -.08, [-.21, .06]

		Measured **before** extreme behavioral willingness				what extent would you be willing to fight them?	
					Commit violence	To what extent would you be willing to engage in violence on behalf of your position on mask wearing requirements?	*r*(207) = .11, [-.03, .24]
					Self-isolation (COVID)	To what extent are you willing to completely isolate yourself from all other people you do not live with until the pandemic is over to help prevent the spread of COVID-19?	*r*(207) = -.29, [-.41, -.16]
					Get opponents fired	To what extent would you be willing to try to get someone who disagrees with you about mask wearing requirements fired from their job?	*r*(207) = -.10, [-.23, .03]
				Moderate	Wear mask (COVID)	To what extent are you willing to wear a mask in public to help prevent the spread of COVID-19?	*r*(207) = -.38, [-.49, -.25]
					Advocate	To what extent are you willing to try to persuade others who disagree with you about mask wearing requirements?	*r*(207) = -.46, [-.56, -.35]
					Political support	To what extent are you willing to support political candidates who share your views on mask wearing requirements?	*r*(207) = -.37, [-.48, -.25]

					Avoid crowds (COVID)	To what extent are you willing to avoid large crowds of people to help prevent the spread of COVID-19?	$r(207) = -.42$, [.52, -.30]
12	Political orientation	"…about your political beliefs and opinions?<br><br>Measured **after** extreme behavioral willingness	252	Extreme	Fight opponents	If someone was behaving in a way that is opposed to your political beliefs and opinions, to what extent would you be willing to fight them?	$r(250) = .36$, [.25, .47]
					Commit violence	To what extent would you be willing to engage in violence on behalf of your political beliefs and opinions?	$r(250) = .34$, [.23, .45]
					Confront opponents	To what extent would you be willing to aggressively confront someone who was acting in a way that is opposed to your political approach?	$r(250) = .26$, [.14, .37]
					Argue with opponents	To what extent would you be willing to get into a heated argument with someone if they attacked your political beliefs and opinions?	$r(250) = .14$, [.01, .25]
				Moderate	Vote	To what extent would you be willing to vote for political candidates who share your political beliefs and opinions?	$r(250) = -.33$, [-.44, -.22]
					Advocate	To what extent would you be	$r(250) = -.10$, [-.22, .02]

						willing to advocate to others on behalf of your political beliefs and opinions?	
					Read news	To what extent would you be willing to read newspaper articles that support your political beliefs and opinions?	*r*(250) = -.08, [-.20, .04]
			251		Donate money	To what extent would you be willing to donate money to causes that support your political beliefs and opinions?	*r*(249) = .03, [-.09, .15]
13	Political orientation	"…about your political beliefs and opinions?"<br><br>Measured **after** extreme behavioral willingness	203	Extreme	Fight opponents	If someone was behaving in a way that is opposed to your political beliefs and opinions, to what extent would you be willing to fight them?	*r*(201) = .35, [.22, .47]
					Commit violence	To what extent would you be willing to engage in violence on behalf of your political beliefs and opinions?	*r*(201) = .51, [.40, .61]
					Confront opponents	To what extent would you be willing to aggressively confront someone who was acting in a way that is opposed to your political approach?	*r*(201) = .30, [.17, .42]
				Moderate	Vote	To what extent would you be willing to vote for political candidates	*r*(201) = -.40, [-.51, -.28]

						who share your political beliefs and opinions?	
					Advocate	To what extent would you be willing to advocate to others on behalf of your political beliefs and opinions?	*r*(201) = -.16, [-.29, -.02]
					Display views	To what extent would you be willing to publicly advertise your political beliefs and opinions (e.g., via social media, a bumper sticker, t-shirt, etc.)?	*r*(201) = -.10, [-.24, .04]
14	Political orientation	"…about your political beliefs and opinions?"<br><br>Measured **after** extreme behavioral willingness	349	Extreme	Fight opponents	If someone was behaving in a way that is opposed to your political beliefs and opinions, to what extent would you be willing to fight them?	*r*(347) = .29, [.19, .38]
					Commit violence	To what extent would you be willing to engage in violence on behalf of your political beliefs and opinions?	*r*(347) = .37, [.28, .46]
				Moderate	Vote	To what extent would you be willing to vote for political candidates who share your political beliefs and opinions?	*r*(347) = -.22, [-.32, -.12]
					Advocate	To what extent would you be willing to advocate to others on behalf of your political	*r*(347) = -.03, [-.13, .08]

						beliefs and opinions?	
					Donate money	To what extent would you be willing to donate money to causes that support your political beliefs and opinions?	*r*(347) = -.07, [-.03, .18]
					Put in effort	To what extent would you be willing to put effort into supporting your political beliefs and opinions?	*r*(347) = -.07, [-.17, .04]
					Spend time	To what extent would you be willing to spend time supporting your political beliefs and opinions?	*r*(347) = -.07, [-.17, .04]
				N/A	Make sacrifices	To what extent would you be willing to make sacrifices on behalf of your political beliefs and opinions?	*r*(347) = .08, [-.03, .18]
15	Political orientation	"…about your political beliefs and opinions?"<br><br>Measured **after** extreme behavioral willingness	211	Extreme	Fight opponents	If someone was behaving in a way that is opposed to your political beliefs and opinions, to what extent would you be willing to fight them?	*r*(209) = .16, [.02, .29]
					Violence	To what extent would you be willing to engage in violence on behalf of your political beliefs and opinions?	*r*(209) = .18, [.04, .31]
				Moderate	Vote	To what extent would you be willing to vote for	*r*(209) = -.25, [-.37, -.12]

						political candidates who share your political beliefs and opinions?	
					Advocate	To what extent would you be willing to advocate to others on behalf of your political beliefs and opinions?	*r*(209) = -.20, [-.33, -.07]
					Donate money	To what extent would you be willing to donate money to causes that support your political beliefs and opinions?	*r*(209) = -.12, [-.25, .02]
					Put in effort	To what extent would you be willing to put effort into supporting your political beliefs and opinions?	*r*(209) = -.20, [-.32, -.06]
					Spend time	To what extent would you be willing to spend time supporting your political beliefs and opinions?	*r*(209) = -.20, [-.33, -.07]
				N/A	Make sacrifices	To what extent would you be willing to make sacrifices on behalf of your political beliefs and opinions?	*r*(209) = -.03, [-.17, .10]
16	Political orientation	"…about your political beliefs and opinions?"<br><br>Measured **after** extreme	209	Extreme	Fight opponents	If someone was behaving in a way that is opposed to your views about political beliefs and opinions, to what extent would you be willing to fight them?	*r*(207) = .41, [.29, .52]

		behavioral willingness			Commit violence	To what extent would you be willing to engage in violence on behalf of your political beliefs and opinions?	*r*(207) = .48, [.37, .58]
				Moderate	Vote	To what extent would you be willing to vote for political candidates who share your political beliefs and opinions?	*r*(207) = -.33, [-.45, -.21]
					Advocate	To what extent would you be willing to advocate to others on behalf of your political beliefs and opinions?	*r*(207) = -.17, [-.30, -.04]
					Donate money	To what extent would you be willing to donate money to causes that support your political beliefs and opinions?	*r*(207) = -.01, [-.14, .13]
					Display views	To what extent would you be willing to publicly advertise your political beliefs and opinions (e.g., via social media, a bumper sticker, t-shirt, etc.)?	*r*(207) = .08, [-.06, .21]
17	Covid precautions	"…about taking recommended COVID-19 precautions?"<br><br>Measured **before** extreme	288	Extreme	Fight opponents	If someone was behaving in a way that is opposed to your views about taking recommended COVID-19 precautions, to what extent would you be willing to fight them?	*r*(286) = -.07, [-.19, .04]

		behavioral willingness			Commit violence	To what extent would you be willing to engage in violence on behalf of your position on taking recommended COVID-19 precautions?	*r*(286) = .21, [.10, .32]
					Self-isolation (COVID)	To what extent are you willing to completely isolate yourself from all other people you do not live with until the pandemic is over to help prevent the spread of COVID-19?	*r*(286) = -.26, [-.36, -.15]
				Moderate	Wear mask (COVID)	To what extent are you willing to wear a mask in public to help prevent the spread of COVID-19?	*r*(286) = -.39, [-.48, -.28]
					Avoid crowds (COVID)	To what extent are you willing to avoid large crowds of people to help prevent the spread of COVID-19?	*r*(286) = -.38, [-.47, -.28]
18	Covid precautions	"…about taking recommended COVID-19 precautions?"<br><br>Measured before extreme behavioral willingness	385	Extreme	Confront opponents	To what extent would you be willing to confront someone who was not complying with recommended COVID-19 precautions?	*r*(383) = -.13, [-.23, -.03]
					Fight opponents	If someone was behaving in a way that is opposed to your views about Covid precautions, to what extent would you be	*r*(383) = .07, [-.03, .17]

						willing to fight them?	
					Commit violence	To what extent would you be willing to engage in violence on behalf of your position on taking recommended COVID-19 precautions?	*r*(383) = .20, [.10, .29]
					Self-isolation (COVID)	To what extent are you willing to completely isolate yourself from all other people you do not live with until the pandemic is over to help prevent the spread of COVID-19?	*r*(383) = -.09, [-.19, .01]
				Moderate	Wear mask (COVID)	To what extent are you willing to wear a mask in public to help prevent the spread of COVID-19?	*r*(383) = -.25, [-.34, -.15]
					Avoid crowds (COVID)	To what extent are you willing to avoid large crowds of people to help prevent the spread of COVID-19?	*r*(383) = -.29, [-.37, -.19]
19	Abortion legality	"…about legal abortion?"<br><br>Measured **before** extreme behavioral willingness	456	Extreme	Fight opponents	*Same items as Study 1, referring to [pro-choice/pro-life] position*	*r*(454) = .11, [.02, .20]

Table S1. Study 2 measures and results by sample. The behavioral willingness measures included in each sample and their relationships with subjective ambivalence. The object of ambivalence (e.g., about one's political beliefs) and measurement order (ambivalence vs. extreme behavioral willingness) are also specified.
